# Methods for independently manipulating palatability and color in small insect prey

**DOI:** 10.1371/journal.pone.0231205

**Published:** 2020-04-07

**Authors:** Alex M. Winsor, Malika Ihle, Lisa A. Taylor

**Affiliations:** 1 Entomology and Nematology Department, University of Florida, Gainesville, FL, United States of America; 2 Organismic and Evolutionary Biology, University of Massachusetts Amherst, Amherst, MA, United States of America; 3 Florida Museum of Natural History, University of Florida, Gainesville, FL, United States of America; Durham University, UNITED KINGDOM

## Abstract

Understanding how the psychology of predators shapes the defenses of colorful aposematic prey has been a rich area of inquiry, with emphasis on hypothesis-driven experiments that independently manipulate color and palatability in prey to examine predator responses. Most of these studies focus on avian predators, despite calls to consider more taxonomically diverse predators. This taxonomic bias leaves gaps in our knowledge about the generalizability of current theory. Here we have adapted tools that have been successfully used with bird predators and scaled them down and tested them with smaller predators (*Habronattus* jumping spiders) and small insect prey (termites, milkweed bug nymphs, pinhead crickets, fruit flies). Specifically, we test (1) the application of denatonium benzoate (DB) to the surface of live termites, crickets, and fruit flies, and (2) the effectiveness of manipulating the palatability of milkweed bug nymphs through diet. We also test the effectiveness of combining these palatability manipulations with various color manipulations. Across several experiments, we confirm that our palatability manipulations are not detectable to the spiders before they attack (i.e., they do not produce aversive odors that spiders avoid), and show that unpalatable prey are indeed quickly rejected and spiders do not habituate to the taste with experience. We also investigate limitations of these techniques by assessing possible unintended effects on prey behavior and the risk of contact contamination when using DB-treated prey in experiments. While similar tools have been used to manipulate color and palatability with avian predators and relatively large insect prey, we show how these techniques can be effectively adapted for use with small invertebrate predators and prey.

## Introduction

Many organisms use conspicuous warning signals to advertise their unpalatability [1, 2]. In particular, bright and conspicuous coloration is often paired with distastefulness, presumably because it heightens the capacity for predator learning [3–6] and reduces the chance that a predator will misidentify prey [7]. Understanding how such prey defenses and warning colors evolve requires substantial consideration of the perceptual and cognitive abilities of the predators that drive their evolution [8–11]. Attention to this idea has led to hundreds of studies examining how the psychology of key predators has shaped the defenses of colorful aposematic insect prey [2]. Much of the insight in this field has been gained by robust and cleverly designed, hypothesis-driven experiments that have independently manipulated color and palatability in prey to examine predator responses [12–15]. Such experiments have been critical in building a rich theoretical framework, yet the vast majority of these studies have been limited to avian predators [2], leaving questions about how generalizable our current theory is to the many other predators that feed on aposematic prey.

Recent studies have emphasized the importance of considering a more diverse set of predators in this work [16,17], with a particular emphasis on considering terrestrial invertebrate predators [18–24], and such studies are revealing surprising insights. In some cases, there are remarkable similarities between arthropod predators and birds; for example, similar (but not identical) patterns of color aversion, learning, and generalization have been shown in jumping spiders [19–21, 24], mantids [25], and predatory wasps [26, 27]. Yet, there are also remarkable differences in the responses of some of these predators. For example, jumping spiders have limited ability to retain learned aversions compared with birds [20], and mantids will readily feed on aposematically colored harlequin bugs that are noxious to birds [18]. Collectively, these results highlight the novel and unexpected insights that can be gained from examining diverse predators.

There are additional reasons that terrestrial invertebrate predators, in particular, are important to consider in this work. A variety of sensory capabilities are required to detect many aposematic signals and prey defenses, and these capabilities differ dramatically between birds and invertebrate predators. The evolution of the avian visual system, including color vision, has been relatively constrained [28] compared with the tremendous diversity of photoreceptor spectral sensitivities that have evolved in insects [29, 30], as well as other arthropods [31]. Many invertebrate predators also have tiny brains compared to birds, but complex and varied cognitive abilities [32]. Given this diversity, aposematic signals will likely be perceived and processed differently depending on the predator that encounters them. In some cases, invertebrate predators feed on the same prey types as birds [33, 34] but also likely feed on smaller prey that birds often disregard [35]. Additionally, the hunting strategies of invertebrate predators are incredibly diverse [36] in ways that differ from birds, which will influence how these predators find and interact with aposematic prey. Importantly, invertebrate predators, because of their large numbers, can effectively regulate prey density in both natural and managed ecosystems [37], and therefore likely exert a strong influence on the evolution of their color patterns and defenses.

Our goal in the present study was to develop a set of tools that will encourage more manipulative experiments using small terrestrial invertebrate predators in the study of aposematism. Many tools for independently manipulating palatability and color have proven successful in studies of bird predators; here we have adapted and tested some of these tools for use with smaller predators (*Habronattus pyrrithrix* jumping spiders*)* and small insect prey (termites, milkweed bug nymphs, pinhead crickets, fruit flies). Among invertebrate predators, jumping spiders (Family Salticidae) are an excellent group to study these phenomena, as they are voracious predators [38], demonstrate complex decision making and learning during foraging [39], and are common worldwide, found on all continents other than Antarctica [40]. Jumping spiders in the genus *Habronattus* can discriminate long-wavelength colors (e.g. yellow, orange, red) [41] common in aposematic signals. Thus, attention to the profitability of color is likely beneficial in a foraging context. Indeed, *Habronattus* and other jumping spider species have already provided useful insights into predator psychology and the evolution of aposematism [19–21, 23, 24].

Here, we describe and test the effectiveness of two methods to manipulate the palatability of small insect prey and present various methods to manipulate prey color that can be used in conjunction with these palatability manipulations. First, we test the application of denatonium benzoate (hereafter referred to as ‘DB’, also known under the trade name Bitrex®) to the surface of termite, cricket, and fruit fly prey. Second, we test the effectiveness of manipulating the palatability of milkweed bug nymphs by feeding them different diets (milkweed vs. sunflower seeds). Across several experiments, we assess whether the prey palatability manipulations are detectable to the spiders before they attack (i.e., to confirm that the palatability manipulations do not produce aversive odors that spiders might initially avoid, or learn to avoid), and whether, once attacked, unpalatable prey are indeed quickly rejected (and that spiders do not habituate to the taste with experience). We also investigate possible limitations of these techniques by comparing the movement rate of manipulated prey to control prey (to assess unintended effects on prey behavior) and by assessing the risk of contact contamination between DB-treated and control prey used in our experiments. By validating multiple methods to independently manipulate palatability and color in these small predators and prey, we aim to establish a robust set of tools for behavioral ecology research. While similar tools have been previously used to manipulate color and palatability with avian predators and relatively large insect prey such as mealworms [42], adult firebugs [43], and butterflies [44], we show here that these techniques can be effectively modified and scaled down for use with small invertebrate predators and prey that are a fraction of their size.

## Materials and methods

To maximize the objectivity of the presented research, we preregistered the study hypotheses, protocols, sample sizes, and data analyses (hereafter ‘confirmatory analyses’) for experimental sets 1 and 2 before the start of data collection [45, 46] (see [47] for the rationale behind preregistration). We closely adhered to our statistical plan and performed additional analyses (hereafter ‘exploratory analyses’), all presented below.

### Data availability

All collected data, analysis scripts, and workflow are archived and publicly available in the Open Science Framework [48] and can be accessed here: https://doi.org/10.17605/OSF.IO/JSPW3.

### Collection and maintenance of spiders

We collected juvenile female *Habronattus pyrrithrix* between May and July 2018, from a single population in Queen Creek, Arizona, USA (33°13′29″N, 111°35′34″W). No permits were required as all spiders were collected from private property with the permission of the property owners (Schnepf Farm). These spiders were reared and maintained in the laboratory using previously described methods [19] until reaching maturity. Briefly, spiders were maintained in transparent plastic boxes (58 x 58 x 129mm) fitted with a mesh top and an artificial green plant (approximately 10 cm long, Ashland Fern Collection, Michael's Stores, Irving, TX, USA) for enrichment. All boxes were housed in a climate-controlled room (24.5°C, 14/10-hour light/dark cycle). Spiders were fed a diet of crickets (*Gryllodes sigillatus*) and fruit flies (*Drosophila melanogaster*) 3 times per week until entering an experiment. We used female spiders only in our experiments, as females are typically more motivated to feed compared with males and juveniles [19].

Because color vision in *Habronattus* jumping spiders appears to be light-limited [49, 41], spiders were maintained and fed under full-spectrum artificial lights (SoLux MR16 3500K 50W, Tailored Lighting Inc, Rochester, NY, USA) supplemented with natural sunlight. We conducted all tests (described below) in the laboratory under full spectrum bulbs (SoLux PAR38 3500K 90W, Tailored Lighting Inc, Rochester, NY, USA), directly adjacent to large windows during daylight hours (between 0900 and 1700 hours).

### Collection of prey

Worker termites (*Reticulitermes flavipes*) were extracted from decaying logs at the University of Florida Natural Area Teaching Laboratory (29°38'02.6"N 82°22'06.0"W). Milkweed bug nymphs (*Oncopeltus fasciatus*) were obtained from two distinct laboratory colonies (described later in Methods) originating from specimens purchased from Carolina Biological Supply Company (Burlington, NC, USA). Colonies of crickets (*Gryllodes sigillatus*) and fruit flies (*Drosophila melanogaster*) were initially obtained from Top Hat Cricket Farm (Portage, MI, USA) and Carolina Biological Supply Co. respectively, and were subsequently reared in the laboratory.

### Experimental set 1: DB-induced unpalatability in termites

The first experimental set investigated the effectiveness of applying DB to the surface of termites to make them unpalatable to spiders. DB is a non-toxic and highly ‘bitter’ compound (see [50, 51] for a discussion of bitterness in the context of animal gustation), which is colorless, supposedly odorless, and has been used in several previous studies to manipulate the palatability of the external surface of prey given to predominantly avian predators [52–56, 14]. While quinine has long been used as a bittering agent in taste aversion studies with many animals [57–59], we chose to use DB here because quinine has been shown to be ineffective with at least some jumping spider species, which will readily eat quinine-laced prey [60]. In invertebrate predators, there are two main aversive conditioning studies (to our knowledge) that used DB, both of which manipulated the palatability of prey offered to mantids by injecting and coating prey with DB solution [61,62]. Despite its widespread effectiveness in studies with avian predators, and more recent use with mantid predators, little is known about the effectiveness of DB when applied solely to the surface of much smaller prey and offered to smaller invertebrate predators.

The goal of this set of experiments was to answer seven specific questions about the effectiveness of DB when applied to prey: (1.1) Does DB treatment unintentionally influence termite movement rate? (1.2) Are there aversive odors associated with DB-treated termites that naïve spiders avoid? (1.3) Are there aversive odors associated with DB-treated termites that experienced spiders can learn to avoid? (1.4) Is DB effective at making termites unpalatable to naïve spiders? (1.5) Do spiders habituate to the taste of DB (i.e., does it become less effective with experience)? (1.6) Does increasing DB concentration increase its effectiveness at making termites unpalatable to spiders? And (1.7) Is there a risk of contamination between DB-treated and control termites when used simultaneously in an experiment? (i.e., could DB be transferred from treated to control termites during an experiment)?

#### Manipulating palatability of termites using DB application

To address the questions above, we first developed a method of applying DB to the exterior surface of termites. Here we used DB powder (Chemsavers, Bluefield, VA, USA) dissolved in distilled water to create solutions of various concentrations, ranging from 1–3% to be used across several separate experiments (described in more detail below). We applied DB solution directly to the entire exterior surface of termites using a spray bottle and allowed them to thoroughly dry (for at least 5 minutes following spraying). Termites sprayed with DB are hereafter referred to as ‘DB termites’ for simplicity. Control termites used in experiments were sham-treated by spraying them with distilled water. Since DB is presumably extremely bitter even at low concentrations, all instruments that came into contact with DB were kept isolated, and all applications of DB to prey were done either outdoors or in a separate well-ventilated room.

#### Termite color manipulation

Because the DB solution is colorless, we needed a way to keep track of which termites in each experiment were DB-treated and which were controls. To do this, we randomly assigned each termite a particular color using a previously published method of termite color manipulation that uses flat oil-based enamel paints (Testors, Rockford, IL, USA) [23, 24]. In this study, we were not interested in the spiders’ color preferences *per se*, and so we strategically chose two colors that we expected to be neutral to the spiders and that they would readily attack: green (product #1164TT) and brown (product #1166TT). Leafhoppers displaying these two colors appeared to be the dominant prey available at our field collection site (AMW, personal observation) and while these spiders do exhibit color biases for and against some colors in foraging (e.g., against red and yellow, and for blue), we had no *a priori* reason to expect differences in spider responses to green or brown [19]. Spiders were exposed to both of these colors in the lab (outside of the context of foraging); they had a green plant affixed to the side of their box (described above) and our lab feeding table was brown and visible through the clear floor of their container. Because prior empirical evidence shows that contrast with the background can influence the conspicuousness of prey, and therefore predator responses [25], we adjusted the brightness of our green and brown paints to be as similar as possible to one another (see additional detail below) to prevent one color from being more conspicuous than the other on the white background on which they were presented to the spiders.

To adjust paint brightness, we first used a UV-vis spectrophotometer (USB 2000+ with PX-2 pulsed xenon light source, Ocean Optics, Dunedin, FL, USA) to measure paint reflectance. All spectral measurements were relative to a Spectralon® diffuse white reflectance standard (Labsphere Inc., North Sutton, NH, USA). Due to oversaturation from our PX-2 light source, artifacts manifested in all measurements at two distinct ranges of the spectra (between λ = 475–495nm and λ = 525–540nm). We removed these artifacts using the function CLRspike in the CLR 1.05 software [63], which was designed for this purpose. Measurements of both colors were recorded in a dark room with the probe perpendicular to the sample, and the emitted light fully covering the colored surface. The brown paint was supplemented with black paint (product #1149TT) until the brightness of the green and brown paints (calculated as the mean reflectance between the range of 280–700 nm, see [64]) were matched (two-tailed t-test, *P* = 0.38, see S1 Fig). Our analyses focused on the range of 280–700nm because this is thought to be the wavelength range visible to jumping spiders [65,66].

**Fig 1:**
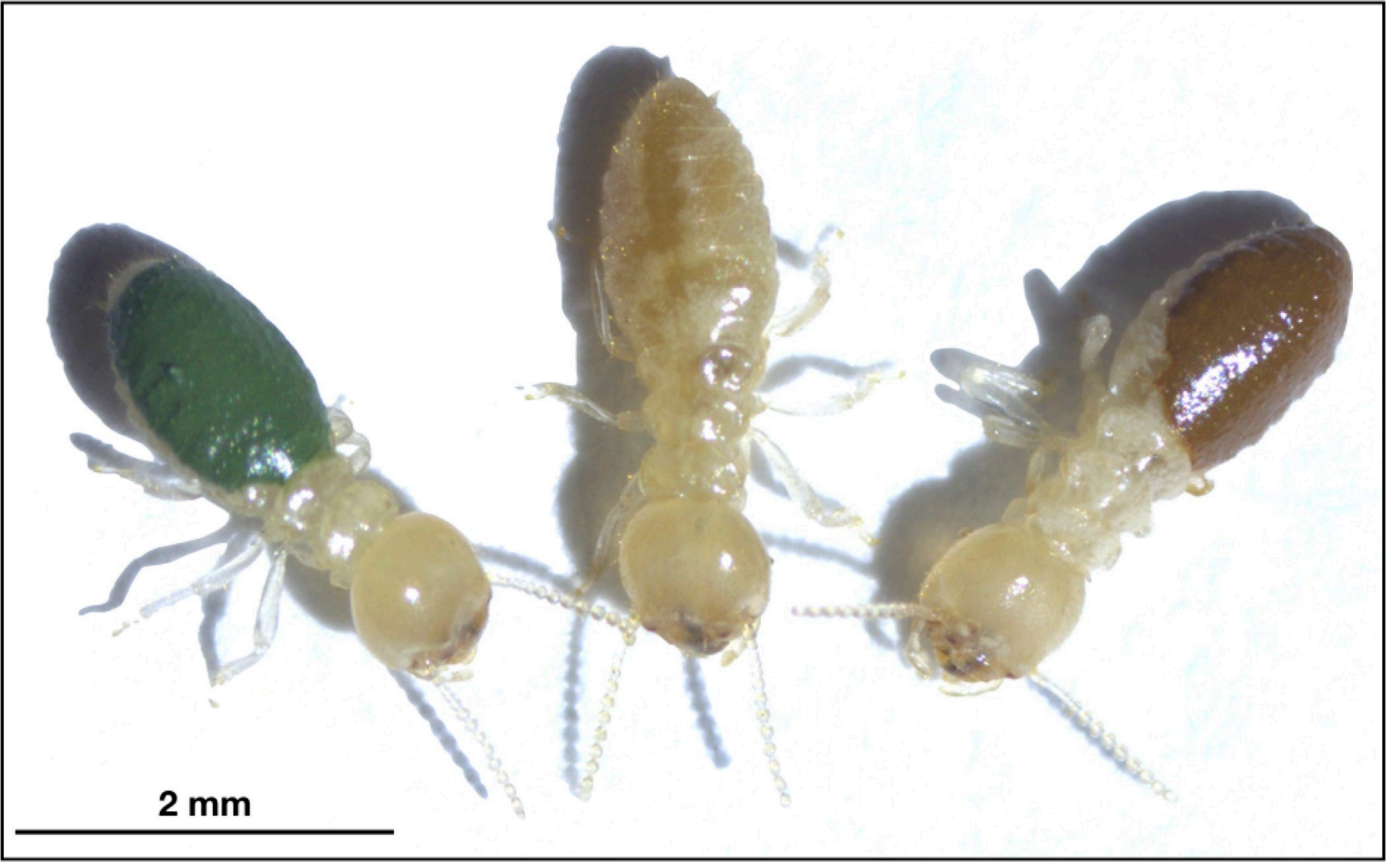
Artificially colored termites (*Reticulitermes flavipes*) painted on the dorsal surface of their abdomens with enamel paint for use in prey choice experiments (Experimental set 1). Green-painted (left), unpainted (center), and brown-painted (right) termites are shown for comparison.

Paint was applied to the termites with a small pointed tip paint brush (Testors, Rockford, IL, USA), only on the dorsal side of the abdomen (Fig 1). During the painting process, termites were freely roaming a petri dish with a filter paper substrate. If paint spread beyond the abdomen (e.g., onto the legs or thorax), the termite was discarded. Previous studies revealed no significant differences in movement rates between unpainted and painted termites [23,24] suggesting that this color manipulation does not inhibit their natural movement rates.

#### Assessing whether DB treatment unintentionally influences termite movement rate

We compared movement rates between DB-treated and control (sham-treated) termites while they were simultaneously roaming in a petri dish (100 x 15mm) lined with filter paper with printed gridlines (squares with a length/width of 3mm). Each trial included two termites (one treated with 3% DB and one control). To keep track of which termite was DB-treated and which was the control, for each trial we randomly assigned each termite to be painted either brown or green. Termites were placed into the arena exactly 10 minutes after being painted, and 5 minutes after being sprayed (with either DB or water). Our total sample size was n = 120 termites (n = 30 for each of the four possible color-palatability combinations). To measure movement rate, we counted the number of gridlines crossed by each termite. A countable gridline cross was defined as when >50% of the termite’s body extended over a line. Trials lasted 10 minutes and were video recorded, with the final 2 minutes subject to analysis. Because color was assigned to treatments at random (and assignments were different for each trial), observers collecting data from the videos were blind to the DB treatment of the termites.

#### Experimental design for choice tests between DB-treated and control termites (Experiments A and B)

We ran two large multi-part experiments to test the effectiveness of using DB to make termites unpalatable to spiders (hereafter referred to as Experiment A and Experiment B). In Experiment A, we used only spiders that were naïve to DB (see additional details below). In Experiment B, we used a different subset of spiders, half of which were naïve to DB and half of which were intentionally exposed to DB just before their tests.

The goal of Experiment A was to assess how naïve spiders would respond to DB-treated termites. In Experiment A, all tested spiders had been used in a single separate experiment also involving DB, but had not experienced DB for at least two weeks prior to testing (which previous work suggests is a sufficient time frame for jumping spiders to forget a training stimulus, see [67, 20]). Hereafter, we will refer to these spiders as “naïve”. With these naïve spiders, we first ran choice tests to ask whether they would avoid attacking DB-treated termites (compared to controls), as would be expected if they were responding to aversive odors present in the DB that they could detect prior to attack. We also recorded prey rejection rates (i.e., if a prey item was dropped within 2 minutes of attack), which allowed us to determine if our DB treatment was successfully making the termites unpalatable to these naïve spiders. Within Experiment A, choice tests were replicated across three concentrations of DB (1.5, 2, and 3%) to allow us to assess whether there were consistent patterns across these concentrations and whether increasing concentration increased the effectiveness of DB at making termites unpalatable. We tested n = 90 spiders in Experiment A (n = 30 spiders at each DB concentration).

The goal of Experiment B was to assess how experienced spiders would respond to DB-treated termites and whether this differed from the responses of naïve spiders. Even if naive spiders do not initially discriminate between the odor of DB-treated and untreated prey (as we found in Experiment A, see Results), repeated experience with an odor could potentially trigger the spider to respond to it in future encounters (i.e., the olfactory cues might be detected but ignored by spiders with no prior exposure to such a stimulus). Likewise, even if naïve spiders readily reject DB-treated prey (as we found in Experiment A, see Results), they may habituate to it with repeated exposure making it ineffective in experiments where the same spiders have to be tested repeatedly. To test these ideas in Experiment B, we conducted identical choice tests to those in Experiment A using a new set of spiders. At the start of the experiment, none of these spiders had ever been exposed to DB solution. These spiders were randomly divided into two treatment groups: one group would be exposed to DB prior to their choice tests, while the other group would remain naïve to DB until their choice tests. For the DB-exposed group, a DB-treated termite was offered to each spider on each of six consecutive days leading up to their choice test. The DB-treated termite was offered to the spider for a period of 15 minutes (allowing the spider to attack it), after which a palatable non-DB termite was added to provide food for the spider and to prevent the spider from associating termites with unpalatability generally. Although we did not systematically track whether spiders attacked the training prey, we know from anecdotal observation (during this and previous experiments) that they almost always attack this type of prey when initially introduced to them. Spiders in the unexposed (‘naïve’) spider group were offered a termite sprayed with distilled water on each of these exposure days. All termites used during this exposure period were offered to the spiders in a small open petri dish (35 x 11mm) lined with white filter paper that was placed in the bottom of the spider’s box. Within Experiment B, choice tests were replicated across two concentrations of DB (1 and 3%); as in Experiment A, this allowed us to assess whether there were consistent patterns across these concentrations and whether increasing concentration increased the effectiveness of DB at making termites unpalatable. We tested n = 100 spiders at the 1% DB concentration and n = 80 spiders at the 3% DB concentration (we used the maximum number of spiders available that were totally naive to DB for this experiment, and due to the death of one spider during the exposure period, the final sample size was n = 79 for the 3% DB); at each concentration, spiders were split evenly across exposure treatment groups (DB-exposed vs. naïve).

#### Details of termite choice tests

The details of the choice tests for Experiments A and B were identical. Two days before the choice tests began, spider feeding was withheld and remaining prey were removed; this food restriction was strategic in that it ensured that all spiders were sufficiently hungry and motivated to feed, but not so hungry that they would simply attack the first termite they saw.

We presented each spider with a choice of two termite types, each having a particular combination of manipulated color and palatability. Spiders were randomly assigned to one of two groups: they were either presented with two DB-treated termites painted green and two control termites painted brown, or two DB-treated termites painted brown and two control termites painted green. The purpose of these two groups was so (1) the observer (blind to prey treatment) could later tell which of the termites was treated with DB and which was not, and (2) so the DB was not always associated with one color over the other.

We presented these termites to spiders within round foraging arenas (100 x 15mm petri dish) lined with white filter paper that provided a uniform background. Before the trial began, spiders were confined for 2 minutes to a smaller acclimation chamber (35 x 11mm petri dish) with a mesh top positioned in the center of the foraging arena, in such a way that the spiders could view but not attack the surrounding termites. When the trial began, the lid of the acclimation chamber was removed, releasing the test spider into the foraging arena where they could choose among the termites. Termites were allowed to roam in the arena for the entire duration of the test. If a termite died (unexpectedly or from spider attack) or became otherwise immobilized, it was rapidly replaced, and the test continued. A trial was completed once a termite was attacked and fed on for at least 2 minutes or when the maximum allocated time of 2 hours was reached. Spiders that attacked termites were allowed to continue to consume them. If a spider failed to attack any termites in 2 hours, the trial was repeated the next day (without feeding in between) until a choice was made, allowing up to 5 attempts before the spider was replaced (criteria defined *a priori*, see preregistration [46]). Each trial was video recorded (Sony HDR-PJ540) for later analysis.

From the videos, we recorded which prey item was attacked first and the delay to this first attack. An attack likelihood of 50/50 for DB vs. control termites would indicate that the spiders were unable to discriminate between DB-treated and control termites before attacking. Because the spiders do not usually make physical contact with prey before attacking, a bias in favor of attacking control termites (compared with DB termites) would suggest that the spiders were likely responding to and avoiding an odor associated with DB. An odor from the DB treatment might also be implicated if, for their first attack, spiders attacked control termites more quickly than DB termites. We also recorded all instances of prey rejection. If attacked prey are more likely to be rejected when treated with DB (in comparison to a control), then we can conclude that our DB treatment is effective at reducing palatability.

#### Assessing the risk of DB contamination in our experiments

To assess the risk of DB contamination among termites within our trials (as control termites might have been contaminated with low levels of DB from treated termites with whom they were roaming in the same arena during the tests), we tested a new set of n = 30 naïve spiders. During these tests, no termites were treated with DB (although termites were still painted and sprayed with water) which allowed us to establish a baseline prey rejection rate. Prey can be rejected or dropped for a variety of reasons other than taste aversion, including mishandling of prey or a poorly executed attack. This baseline prey rejection rate was used as a comparison with our data in the experiments above. If the baseline prey rejection rate established here is similar to the prey rejection rate for control termites in our DB experiments, this would provide little evidence for contamination. However, if the baseline prey rejection rate here is lower than that for the control termites in our experiments (i.e., spiders are less likely to reject termites here than in our DB experiments), this would suggest the possibility of contamination in our experiments. Contamination may be impossible to fully eliminate but understanding the degree to which it may be occurring is important to establish in order to understand the limitations of particular methods when designing experiments.

### Experimental set 2: Milkweed-induced unpalatability in bugs

The second experimental set investigated the effectiveness of using milkweed bugs that had their palatability manipulated via diet (by feeding them either milkweed seeds to make them unpalatable or sunflower seeds to make them palatable) for use in experiments with small predators. While this technique has been used in several previous studies [68–70, 20, 67], the goal of this set of experiments was to address three remaining questions that are critical to expanding the utility of this method. Specifically, (2.1) do our color and palatability manipulations unintentionally influence bug movement rate? (2.2) Are there aversive odors associated with unpalatable milkweed-fed bugs (compared to sunflower-fed bugs) that spiders can detect prior to attack? (2.3) Is a milkweed diet (in conjunction with our color manipulation) effective at making bugs unpalatable to spiders?

#### Manipulating palatability of bugs using diet

To address these questions, we used previously published methods for manipulating bug diet [20, 67–70]. Milkweed bugs sequester defensive bitter compounds (cardenolide toxins, which are naturally occurring cardiac glycosides) from their diet of milkweed seeds [71]. If milkweed bugs are removed from their natural diet and instead reared on sunflower seeds, which do not contain these compounds, they become palatable to predators [20, 67–70]. In experimental set 2, we used this technique to create third instar milkweed bug nymphs of contrasting palatability. Bugs were reared in laboratory colonies for over 4 years on either milkweed seeds (*Asclepias* sp.) that made them unpalatable or were reared on sunflower (*Helianthus* sp.) seeds to make them palatable. For simplicity, we refer to the milkweed bug nymphs reared on milkweed seeds as milkweed-fed bugs (or MW bugs) and those fed on sunflower seeds as sunflower-fed bugs (or SF bugs).

#### Bug color manipulation

**Fig 2:**
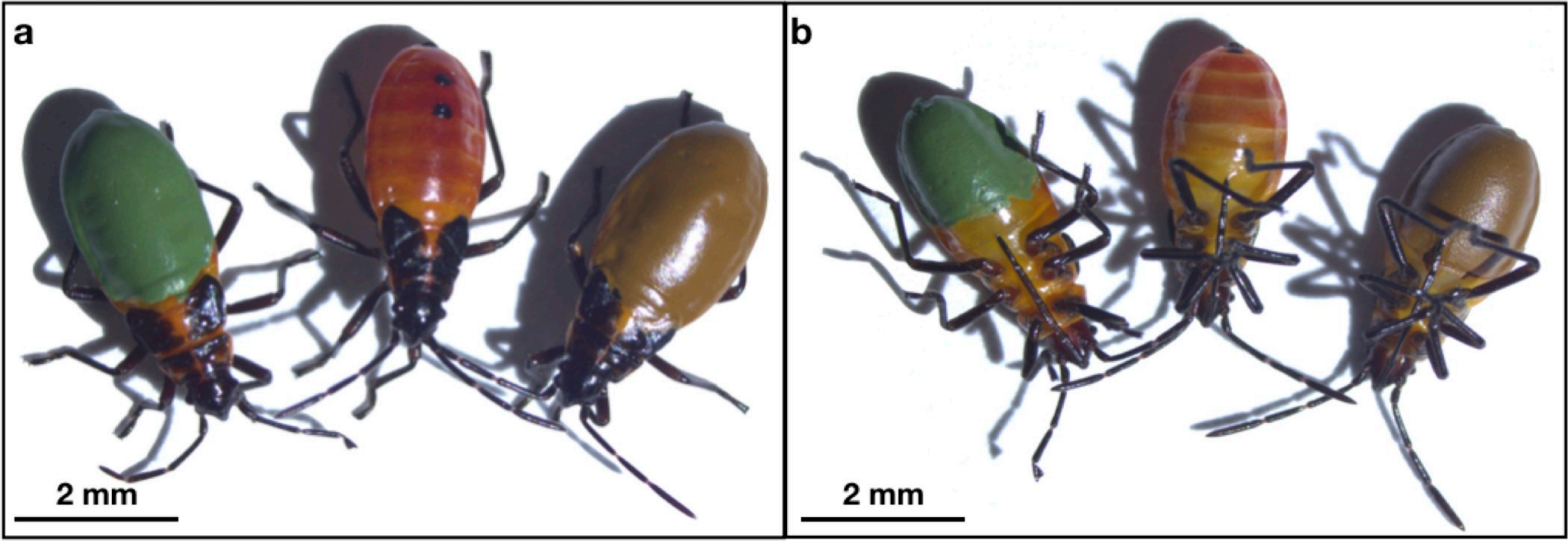
Artificially colored milkweed bug nymphs (*Oncopeltus fasciatus*) painted with enamel paint for use in prey choice experiments (Experimental set 2). (A) Dorsal surface of painted milkweed bugs, showing green-painted (left), unpainted (center), and brown-painted (right) bugs. (B) Ventral surface of painted milkweed bugs, showing green-painted (left), unpainted (center), and brown-painted (right) bugs.

Bugs reared on the two different diets look identical to one another, and thus we needed a way to keep track of which bugs were milkweed-fed and which were sunflower-fed in our experiments. Additionally, bugs climb all surfaces in the spider box so covering up all of their natural red coloration that would be visible to a predator is necessary in an experiment aiming to manipulate the color of aposematic prey. To accomplish this, we combined our method of color manipulation described above for termites (using the same enamel paints) with a method of micro color manipulation, previously developed to paint small areas of jumping spiders such as their faces [72], to coat both the dorsal and ventral portions of the thorax and abdomen (Fig 2A and 2B). In preparation for painting, bugs were anesthetized with carbon dioxide gas for a period of 20 seconds, and promptly mounted to the flat head of a pin using non-toxic water-based glue (Elmer’s school glue, High Point, NC, USA), and positioned under a dissecting microscope connected to a computer and camera (Zeiss Zen, Jena, Germany). Paint was applied uniformly with a microbrush (MicroMark, Berkeley Heights, NJ, USA) first on the underside of the bug using previously described micro-painting techniques [72] with careful attention to avoid any paint application on the legs. After the bug awoke from anesthesia, it was carefully removed from the head of the pin; during this time the bug was still moving slowly which allowed us to paint the dorsal and lateral sides (connecting to the previously painted area). If any of the underlying red coloration was still visible after painting, bugs were either discarded or anesthetized and re-painted.

#### Assessing whether diet treatment and color manipulation unintentionally influence bug movement rate

We compared movement rates between milkweed-fed and sunflower-fed bugs (n = 20 of each, neither of which were anesthetized or painted) to assess the effect of diet treatment; while diet-manipulated milkweed bugs have been used widely in jumping spider foraging experiments [20, 67, 70], none of these prior studies verified a lack of behavioral differences between the two types of bugs. Because this study was the first to use enamel paint to manipulate the color of milkweed bug nymphs, we also wanted to assess if this color manipulation unintentionally influenced bug movement rate; for these tests, we used an additional group of milkweed bugs (n = 20) that were anesthetized and painted green to compare to the unpainted milkweed bugs (n = 20, described above). Movement rates were measured by allowing the bugs to roam individually in a petri dish (100 x 15mm) lined with filter paper with printed gridlines (squares with a length/width of 7mm). Each test began by placing a single bug in the arena exactly 1 hour after waking up from anesthesia. Because the bugs were able to climb the walls of the petri dish, we adjusted the camera to record from directly above, enabling the observer to still count crossed gridlines, even if the bug walked on the underside of the petri dish lid (vertical walls were short, so movement across these walls was disregarded). As with the termite tests described above, we counted the number of gridlines crossed by each bug with a countable gridline cross being defined as when >50% of the bug’s body extended over a line. Trials lasted 10 minutes and were video recorded, with the final 2 minutes subject to analysis. Unlike the termite movement tests above (where movement rates of DB-treated and control termites were assessed simultaneously), it is worth noting that the movement rate of bugs was assessed while they were housed singly in their arenas; this was a practical decision so that we could maximize our sample size to make two separate planned comparisons: (1) unpainted milkweed-fed vs. unpainted sunflower-fed bugs and (2) unpainted milkweed-fed bugs vs. painted milkweed-fed bugs.

#### Experimental design for choice tests between milkweed-fed and sunflower-fed bugs (Experiment C)

The goal of experiment C was to assess the effectiveness of using diet to manipulate milkweed bug palatability. While our tests with termites (Experiment B) revealed no significant effect of a spider’s past experience on their responses to DB-treated prey (see Results), here we chose to expose all spiders to unpalatable milkweed-fed bugs prior to their choice tests. Our rationale for this decision was twofold. First, we wanted to know if there were possible aversive odors associated with milkweed-fed bugs that could be learned and cause spiders to avoid them before attack. Second, because it is possible that spiders might habituate to the taste of the toxins, we wanted to assess if spiders were still deterred with repeated exposure. If spiders were still deterred (and unable to preemptively detect) the toxins even with repeated exposure, then we would expect this manipulation to work with both naïve and experienced spiders. We therefore used n = 40 spiders that were naïve to both SF and MW bugs, and we exposed all of them to milkweed bugs immediately before their choice tests. We accomplished this by offering an unpainted milkweed-fed bug to each spider each day for 6 consecutive days before their choice tests (details of this pre-exposure phase were identical to those used in Experiment B above). During this pre-trial phase, feeding of palatable prey occurred on three non-consecutive days and consisted of two white-eye *Drosophila* given 15 minutes after a new milkweed-fed bug was offered.

#### Details of bug choice tests

In Experiment C, we presented spiders with choices between two bugs, each having a particular combination of manipulated color and palatability. Similar to the termite choice tests, spiders were split randomly into two color groups, with either a MW bug painted green and a control SF bug painted brown, or a MW bug painted brown and a control SF bug painted green. Testing conditions were identical to those used in experimental set 1 (Experiments A and B described above).

As in experimental set 1, we recorded from videos which type of prey was the first to be attacked and measured the delay to that first attack. If there is an aversive odor associated with milkweed-fed bugs, we would expect spiders to attack them at lower rates, or more slowly, compared to sunflower-fed bugs. We also recorded all instances of prey rejection to assess whether the milkweed-fed bugs were rejected at higher rates than controls.

### Experimental set 3: Application of DB treatment to other prey types

The third experimental set investigated the effectiveness of using DB to manipulate the palatability of other prey types in conjunction with other previously published color manipulation techniques. In particular, we examined DB’s effectiveness when applied to termites that were color-manipulated using paper capes [73], pinhead crickets that were colored using food dye [19, 20], and different colored eye mutants of *Drosophila melanogaster*. Considering that our results from Experimental Set 1 showed that DB could be successfully applied to the surface of termites (see Results), our goal here was to ask whether (3.1) DB is effective when applied to other types of color-manipulated prey that are suitable for experiments with small predators. This experimental set features an abbreviated experimental design aimed simply to assess whether our methods are effective with other prey species, in principle.

#### Application of DB to caped termites, dyed crickets, and flies

Because we found evidence that increasing DB concentration increased the effectiveness of making termites unpalatable to spiders (see Results, S3 Fig), we used a 3% DB concentration (the highest concentration used above) for our tests here. Our DB treatments (and sham control treatments) were applied to caped termites and colored crickets in the same way as described above for enamel-painted termites (see details in Experimental Set 1). To apply DB solution to live flies, we contained flies in a clear plastic vial (9 dram, BioQuip, Rancho Dominguez, CA, USA), and carefully sprayed them through a mesh lid.

#### Manipulation of prey color in caped termites, crickets, and flies

**Fig 3:**
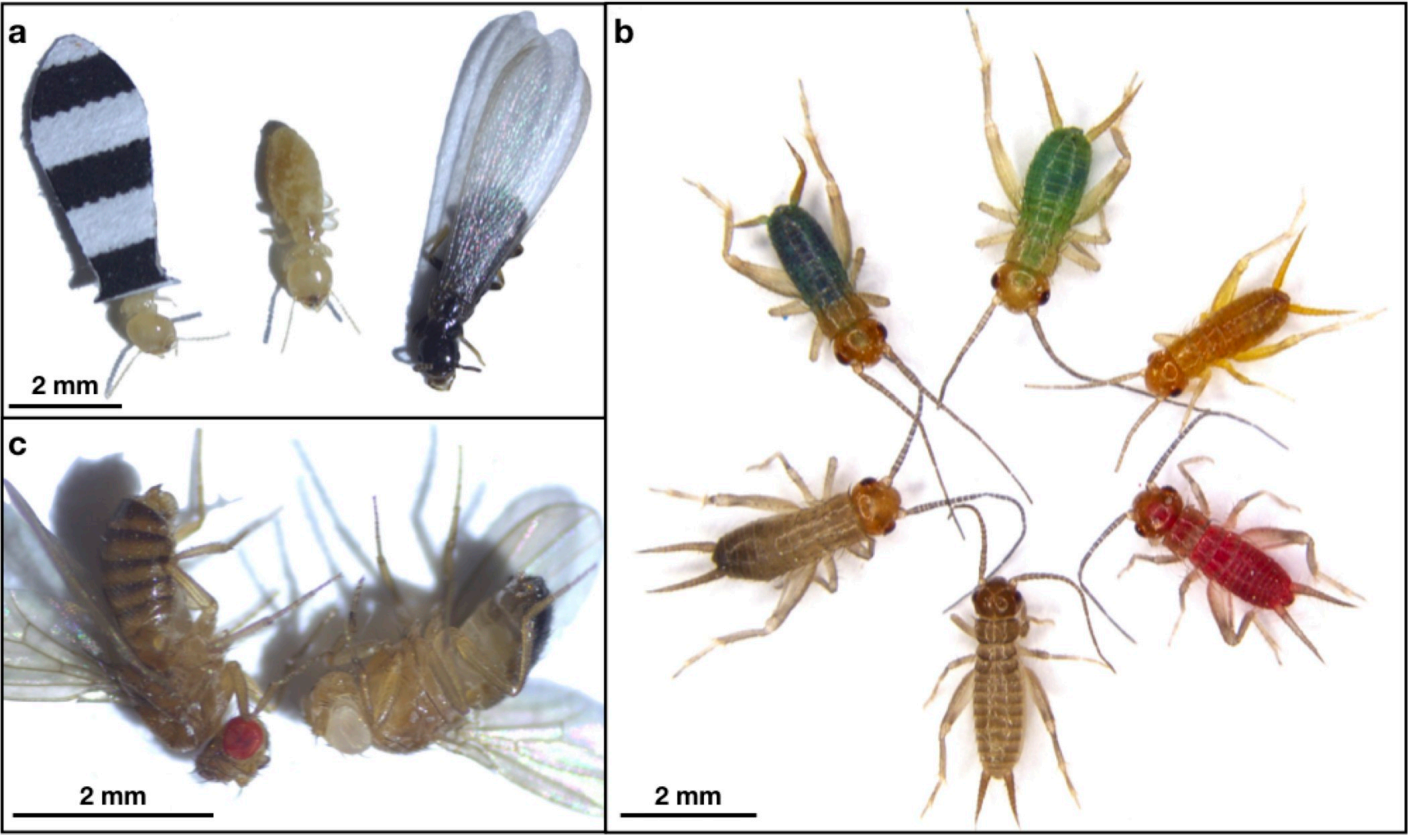
Additional color manipulation techniques used in Experimental set 3. (A) Worker termite (*Reticulitermes flavipes*) with black and white striped cape (left), unmanipulated worker termite (center), and winged termite shown for comparison (right). (B) Color-manipulated crickets (*Gryllodes sigillatus*) colored from ingested food dye. Starting from the top, moving clockwise: green, yellow, red, unmanipulated, brown, and blue (reproduced from [19]; this reference also provides spectral properties of these colored crickets). (C) Fruit flies (*Drosophila melanogaster*) with wild-type red-eye phenotype (left) and mutant white-eye phenotype (right).

To manipulate color patterns in termites, we attached black-and-white striped paper capes to their abdomens using water-based glue (see [73], Fig 3A). To manipulate the body coloration of crickets, we added red food dye to their drinking water (see [19, 20], Fig 3B). To obtain fruit flies with conspicuously different eye colors, we obtained flies from white-eyed mutant and red-eyed wild type genetic lines (Fig 3C).

#### Experimental design for choice tests between DB-treated and control caped termites, colored crickets, and flies (Experiment D)

For DB to be found effective with these alternative color-manipulated prey types, spiders should reject DB-treated prey at higher rates compared to control prey. To investigate this, we presented a new set of spiders (different from Experiment A, B, and C) to either a single DB-treated prey item or to an untreated control and assessed rejection rates in both groups. Aside from the number of prey offered and the duration of tests, which here lasted only 10 minutes, all testing procedures were identical to those described above. Sample sizes for each prey type and treatment group (manipulated or control) were n = 10. Because the 3% DB solution was not effective at making crickets unpalatable to the spiders (see Results), we presented a new set of 20 spiders with crickets treated with a 5% DB solution.

### Statistical analysis

All data processing and analyses were done in R version 3.5.3 [74]. General and generalized mixed effect models described below (LMM and GLMM, respectively) were run using the functions ‘lmer’ and ‘glmer’ from the package lme4 (v1.1-18-1, [75]).

#### Statistical analysis for experimental set 1

*1.1 Does DB treatment unintentionally influence termite movement rate*? To examine if there were differences in movement rates (number of gridlines crossed in test arenas) between control and DB termites (treated with 3% DB), we ran a GLMM with a Poisson distributed error and a log link function, with the DB treatment of the termite (control or DB) and color (painted green or brown) as factors (with brown DB-treated as the intercept), the trial ID as a random effect (as two termites were tested at the same time), and an observation level random effect (i.e., the prey ID) to account for overdispersion [76]. We had no *a priori* reason to expect termite color to be important in this experiment (see above) but we included color in the model to confirm this.

*1.2 Are there aversive odors associated with DB-treated termites that naïve spiders avoid?* If naïve spiders were deterred by an odor from the DB (that they can detect prior to attack), we would expect them to be less likely to attack DB-treated termites compared to controls in Experiment A. As described previously, in each trial, a spider was presented with 2 termites with one color/palatability treatment and 2 termites with the opposite color/palatability treatment. For this analysis, we randomly selected one focal termite type from each trial (i.e. one group of two similarly treated termites). This is because, in tests with two mutually exclusive choices, there is only one informative datapoint (either of the two choices) and keeping both would considerably and artificially inflate the precision of the effect size. We used a generalized linear model (GLM) with a binomial distribution to test whether the focal termite’s palatability treatment (control vs. DB) or color (green vs. brown) predicted whether it would get attacked first in the trial (Y/N). As above, we had no *a priori* reason to expect that spiders would show a specific directional bias between green and brown painted termites, but we included color as a factor to explore this. Because this analysis was based on randomly selecting a focal group of termites, the results may slightly vary depending on which focal termite group was randomly selected from each trial; to account for this, we ran the procedure 1000 times and averaged the analysis results over those 1000 randomly selected subdatasets. We repeated this analysis for the three different DB concentrations used (1.5%, 2%, 3%).

Even if naïve spiders are equally likely to attack control vs. DB termites (as our analyses described above show, see Results), there may still be odors associated with the DB that cause spiders to attack them more cautiously (i.e., more slowly). To explore this idea, we asked whether there was a larger delay (in seconds) before attacking DB termites (compared to attacking control termites) for each spider’s first attack. For this, we used a linear model (LM) to examine whether the palatability treatment (control vs. DB) affected the delay to attack. We repeated this analysis for each concentration of DB used (1.5%, 2%, 3%).

*1.3 Are there aversive odors associated with DB-treated termites that experienced spiders can learn to avoid?* To examine if a spider’s previous experience with DB would make them more likely to respond to any DB-related odors (as might be expected if they learn to be more attentive to such an odor after a negative experience), we asked whether naïve spiders and those that had been previously exposed to DB would differ in their responses to control vs. DB termites (in Experiment B). This analysis was structured the same as the one described above, except here we included prior exposure (naïve vs. DB-exposed) as an additional factor, in interaction with the palatability treatment. In this model, a significant interaction would indicate that naïve and exposed spiders react differently to the presence of DB. If there is no such interaction, the main effect of palatability would indicate whether or not spiders, regardless of their prior exposure, discriminate DB-treated from control termites prior to attacking them. Again, this analysis was repeated at each DB concentration used (1% and 3%).

To determine if prior exposure to DB made spiders more wary to attack DB termites (compared with naïve spiders), we ran a separate model (another LM) to examine whether the palatability treatment of the termite, in interaction with the spider’s prior experience (naïve vs. DB-exposed) affected the delay to attack. Following the same logic described previously, a significant interaction would indicate that naïve and exposed spiders react differently to the presence of DB. We repeated this analysis for each DB concentration used (1% and 3%).

*1.4 Is DB effective at making termites unpalatable to naïve spiders?* If DB is effective at making termites unpalatable to naïve spiders, we would expect DB termites to be more likely to be rejected after an attack compared with control termites in Experiment A. To test this, we used GLMMs with binomial distributions. The independent variables were the termite color (green vs. brown) and the palatability treatment (control vs. DB), and the dependent variable was whether or not the termite was rejected. Because several attacks by the same spider could occur before a termite was consumed within each trial, spider identity was included as a random factor in the model to account for pseudoreplication. We repeated this analysis at all three of the DB concentrations used (1.5%, 2%, 3%).

*1.5 Do spiders habituate to the taste of DB?* Next we explored whether spiders with prior exposure to DB would habituate to it, potentially rendering the DB treatment ineffective if it were used repeatedly with the same spiders over time (using data from Experiment B). To examine this, we used a model structured in the same way as above (GLMM in 1.4) except that we added spider experience (naïve vs. DB-exposed), in interaction with the palatability treatment. A significant interaction would indicate that naïve and exposed spiders differ in how they respond to the presence of DB.

*1.6 Does increasing DB concentration increase its effectiveness at making termites unpalatable to spiders?* To explore whether the probability of rejecting a DB termite increased with the concentration of the DB solution applied, we extracted all attacks on DB termites across both Experiments A and B. We subsetted our data only to attacks towards DB prey so that we could ask if DB prey, once attacked, are more likely to be rejected with higher DB concentrations. For this, we ran a GLMM with a binomial distribution, using the concentration of the solution as a continuous explanatory variable with a quadratic term (added after visually inspecting the model residuals), spider identity as a random effect to account for pseudoreplication, and whether or not a termite was rejected as the dependent variable.

*1.7 Is there a risk of contamination between DB-treated and control termites when used simultaneously in an experiment?* Here we wanted to examine the idea that control termites used in our experiments (supposedly free of DB) might unintentionally become contaminated with DB during our trials while both control and DB termites were freely roaming in the test arenas. To assess this, we qualitatively compared the baseline rejection probability of sham-treated termites (when no DB contamination was possible) to the data collected from across each of our experiments that involved the use of DB-treated termites (Experiments A and B). If control termites in our experiments were getting contaminated with DB, we would expect the rejection probability of control termites in our experiments to be higher than the baseline rejection probability.

In addition to the contamination that could result from control and DB termites interacting with one another in the test arenas, it is also possible that the spiders’ mouthparts or legs could become contaminated with DB after attacking a DB termite. Alternatively, it may be that spiders were more cautious in general because they had a negative experience with prey. In either case, we would expect that a spider would be more likely to drop a control termite that was attacked after attacking a DB termite (compared with control termites that were attacked first). Using Fisher’s exact tests, we compared the rejection probability of control termites that were either attacked first or attacked after an initial attack on a DB termite. We repeated this exploratory analysis for each of our termite experiments.

#### Statistical analysis for experimental set 2

*2.1 Do our color and palatability manipulations unintentionally influence bug movement rate*? We wanted to confirm that painted and unpainted bugs behaved similarly (by comparing painted and unpainted MW bugs), and that bugs of different palatability treatments behaved similarly (by comparing unpainted MW and unpainted SF bugs). For this, we examined whether there were differences in movement rates across these groups with a GLMM with a Poisson distribution error and log link function. In this model, the type of bug was the sole fixed effect (with 3 levels, and with the non-painted MW bug as the intercept, being the natural state of the bug), an observation level random effect (i.e., prey ID) was included to account for overdispersion [76], and the number of gridlines crossed was the dependent variable. We applied a post-hoc Tukey test with the function ghlt from the package multicomp [77] to assess differences in the two pairs of interest.

*2.2 Are there aversive odors associated with unpalatable milkweed-fed bugs (compared to sunflower-fed bugs) that spiders can detect prior to attack?* To examine the possibility that bugs sequestering toxins (MW bugs) produce odors that are aversive to the spiders (compared with SF bugs that do not have these toxins), we asked whether, within a trial, MW and SF bugs were equally likely to be attacked first, and whether there were any differences in the delay to attack these two groups. For this, we ran the same models as for the first experimental set on termites (see sections 1.2 and 1.3 above), with the exception of not having prior exposure as a factor (as all spiders in this experimental set were given prior exposure to MW bugs).

*2.3 Is a milkweed diet effective at making bugs unpalatable to experienced spiders?* We could not run a GLMM to examine if MW bugs were more likely to be rejected than SF bugs, as we did in section 1.4 (where we asked a similar question about unpalatability in DB-treated termites); the reason for this is that there was no variation in the dependent variable for one of the factors (all MW bugs were rejected when attacked, see Results). Instead, we ran a Fisher exact test comparing the rejection probability (whether a bug was rejected or not) between the two palatability treatments. We ran a separate Fisher exact test to compare rejection probability between the two color treatments. To avoid pseudoreplication, we only used the first attack of each spider.

#### Statistical analysis for experimental set 3

*3.1 Is DB effective when applied to other types of color-manipulated prey*? To examine if our methods of DB application could be successfully applied to other types of color-manipulated prey species to make them unpalatable to spiders, we compared the rejection rates of DB-treated prey and control (sham-treated) prey (with each prey item presented alone to a different individual spider, Experiment D) using Fisher’s exact tests. We used Fisher’s exact tests rather than mixed effect models because all palatable prey items were always consumed and therefore there was no variation in the dependent variable for one factor level to be analyzed. To avoid pseudoreplication, we only used the first attack of each spider. We repeated this analysis for all three prey types examined: paper-caped termites, artificially dyed red crickets, and red-eyed fruit flies.

#### Blinding and inference criteria

The palatability of each prey item was unknown to the observer watching the videos, and therefore all data were collected blind. We established our inference criteria using *P*-values of 5% (α = 0.05). As preregistered, we used one-tailed tests for hypothesis testing where directional differences were expected (i.e., where we expected DB-treated prey or MW bugs to get rejected at higher rates, see [78] for justifications) and two-tailed tests for all exploratory analyses and hypothesis testing where no differences were predicted (e.g., no effect of prior exposure, no effect of the palatability of the prey on the likelihood of first attack, and no effect of the color of the prey). *P*-values were extracted directly from the model output based on likelihood ratio tests for LMs and LMMs and from Wald Z tests for GLMs and GLMMs.

## Results and discussion

### Experimental set 1: DB-induced unpalatability in termites

#### 1.1 Does DB treatment unintentionally influence termite movement rate?

The application of DB did not appear to influence termite movement rate, as there were no differences in the number of gridlines crossed between control and DB-treated termites (n = 120, *z* = -1.20, *P* = 0.23, S2 Fig). In addition, as expected, there were no differences in movement rates between termites painted either green or brown (n = 120, z = -0.81, *P* = 0.42). Here we chose to assess movement rates with DB-treated and control termites simultaneously in the same arenas; this was a practical decision as this is likely how they would be presented to predators in experiments using our methods. It is possible other subtle aspects of prey behavior might change with DB application and influence predation, so we suggest future studies consider all relevant aspects of behavior and carefully consider the context in which they assess these behaviors.

Establishing that this manipulation does not negatively affect movement rates was an important consideration because prey movement has been shown to influence predatory responses in jumping spiders [79, 80] and likely would influence other predators as well. We advocate for assessing such unintended effects of DB treatment, as we have done here, any time it is applied to a new prey type or used in a new experimental context.

#### 1.2 Are there aversive odors associated with DB-treated termites that naïve spiders avoid?

**Fig 4:**
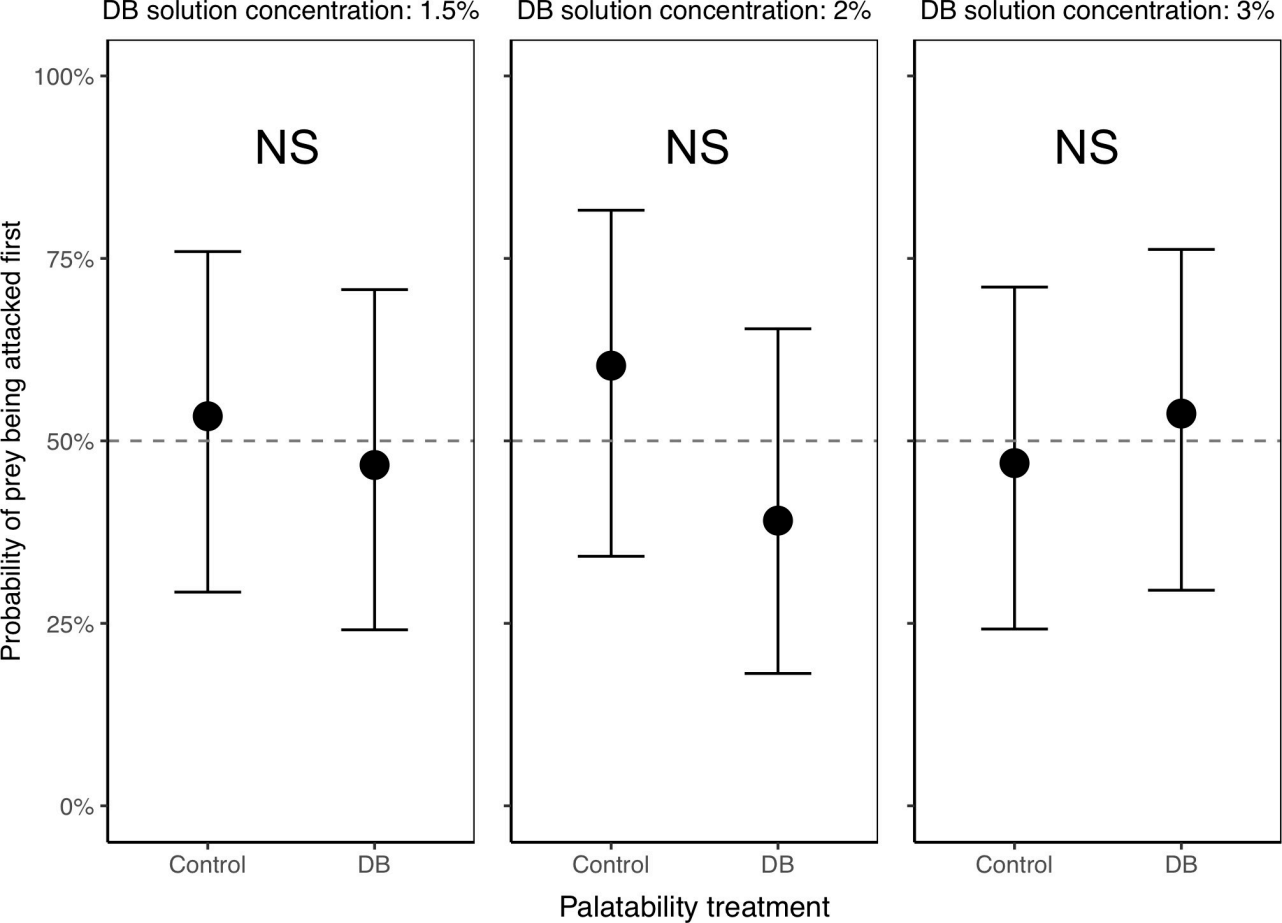
Probability of control vs. DB termites being attacked first, in Experiment A with naïve spiders only. The lack of any significant differences between control and DB termites (at any of the three DB concentrations) suggests that there are no aversive odors associated with our DB treatment that deter attacks from spiders. Plotted are the back-transformed model estimates with their 95% confidence intervals, averaged over 1000 models based on a 1000 random sampling of focal termites. The dotted line at 50% is shown for reference as the expected probability of attack for each group if spiders were not differentiating between control and DB termites. A total of n = 90 spiders were tested (n = 30 at each of the three DB concentrations). NS indicates no significant differences between control and DB termites.

Naïve spiders were equally likely to direct their first attack at a control termite vs. a DB termite for each of the three DB concentrations used (Experiment A, Fig 4 and Table 1), suggesting that regardless of concentration, naïve spiders were not deterred by any odor from the DB. Not surprisingly, there was no significant effect of termite color (green or brown) on their probability of being attacked first (Table 1).

**Table 1:** Model estimates (±SE) of the probability of a particular type of termite being attacked first, in Experiment A with naïve spiders only. Values are relative to the intercept which represents brown control termites. *Z* and *P* values are indicated for each fixed effect. The lack of any significant effects of palatability treatment suggests that there are no aversive odors associated with our DB treatment that deter attacks from spiders.

Factor	Estimate	±SE	*z*	*P*
Experiment with 1.5% DB (n = 30)				
intercept	0.16	0.70	-	-
color (green)	-0.04	0.77	-0.05	0.89
palatability treatment (DB)	-0.29	0.77	-0.37	0.71
Experiment with 2% DB (n = 30)				
intercept	-0.14	0.68	-	-
color (green)	1.22	3.99	1.46	0.15
palatability treatment (DB)	-0.90	0.81	-1.11	0.28
Experiment with 3% DB (n = 30)				
intercept	-0.46	0.68	-	-
color (green)	0.58	0.77	0.74	0.47
palatability treatment (DB)	0.29	0.77	0.37	0.71

Significant *P*-values (when present) are shown in bold.

Likewise, naïve spiders took the same amount of time to attack control and DB termites, for each of the three DB concentrations used (1.5% DB: 270.6 ± 136.5 seconds, Control: 111.2 ± 127.7 seconds, *t* = 0.85, *P* = 0.40; 2% DB: 560.3 ± 360.0 seconds, Control: 833.2 ± 293.9 seconds, *t* = -0.59, *P* = 0.56; 3% DB: 375.8 ± 264.1 seconds, Control: 299.8 ± 282.4 seconds, *t* = 0.20, *P* = 0.85), again suggesting that naïve spiders had no aversion to any odor associated with the DB.

Predators navigate their foraging environment using various sensory inputs for prey detection, including (but not limited to) visual and chemosensory (e.g., olfactory and gustatory) cues [81]. Therefore, it was important to rule out the idea that some predators such as spiders might be able detect a substance like DB, even while it is considered ‘odorless’ to other animals. Our results here could indicate that DB is truly odorless to the spiders, or perhaps that DB-related odors are present and detectable by the spiders, but that they simply do not respond to them. Regardless, any possible odors associated with the DB did not seem to be salient to the spiders in our experiments, making DB an ideal experimental tool for this system. Again, as invertebrate predators have such a diversity of sensory systems that may or may not be able to detect odors associated with DB [82], researchers should consider running similar tests before using DB-treated prey with a new predator species.

Overall, in these experiments with naïve spiders, 93.3% of the spiders attacked a prey item during their first test while 6.7% of the spiders (6 out of 90, 3 at the 2% concentration and 3 at the 3% concentration) needed to be retested (spiders experienced between 1 and 4 tests, median = 1) as they had not made a single attack within the specified 2 hour period (9 spiders were replaced due to meeting exclusion criteria defined *a priori*).

#### 1.3 Are there aversive odors associated with DB-treated termites that experienced spiders can learn to avoid?

**Fig 5:**
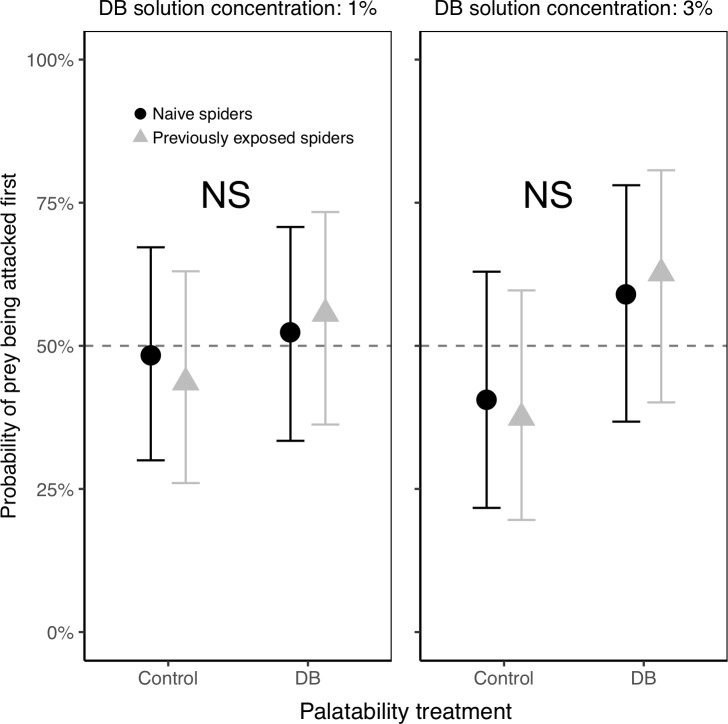
Probability of control vs. DB termites being attacked first, in Experiment B with both naïve spiders and those previously exposed to DB. Naïve spiders and those previously exposed to DB responded similarly; regardless of prior exposure, spiders did not bias their attacks away from DB-treated termites, suggesting that there are no aversive odors associated with DB. Plotted are the back-transformed model estimates with their 95% confidence intervals, averaged over 1000 models based on a 1000 random sampling of focal termites. The dotted line at 50% is shown for reference as the expected probability of attack for each group if spiders were not differentiating between control and DB termites. A total of n = 100 spiders were used for the experiment with 1% DB and n = 79 spiders were used for the experiment with 3% DB. NS indicates no significant differences between control and DB termites.

Spiders which received prior exposure to DB responded to the palatability treatment in the same way as naïve spiders (i.e., there was no interaction between prior exposure and prey palatability treatment across either of the DB concentrations, Experiment B, Fig 5 and Table 2). Spiders were, regardless of their prior exposure, equally likely to direct their first attack towards control vs. DB termites (Fig 5 and Table 2), suggesting that they have neither an innate nor acquired aversion to any odor associated with DB. Again, as expected, termite color (green vs. brown) had no effect on which termite was attacked first (Table 2).

**Table 2:** Model estimates (±SE) of the probability of a particular termite being attacked first, in Experiment B with both naïve spiders and those previously exposed to DB. Values are relative to the intercept which represents naïve spiders that were offered brown control termites. *Z* and *P* values are indicated for each fixed effect. The lack of any significant interaction between palatability treatment and prior DB exposure suggest that both naïve and exposed spiders responded similarly to the DB treatment. The lack of significance of the main effect of palatability treatment suggests that there are no aversive odors associated with our DB treatment that deter attacks from spiders.

Factor	Estimate	±SE	*z*	*P*
Experiment with 1% DB (n = 100)				
intercept	-0.17	0.46	-	-
color (green)	0.17	0.41	0.41	0.69
palatability treatment (DB)	0.16	0.58	0.28	0.77
prior DB exposure (yes)	-0.17	0.58	-0.28	0.58
Interaction between palatability treatment and prior DB exposure (DB*yes)	0.33	0.82	0.40	0.69
Experiment with 3% DB (n = 79)				
intercept	-0.64	0.54	-	-
color (green)	0.50	0.48	1.05	0.30
palatability treatment (DB)	0.77	0.68	1.14	0.26
prior DB exposure (yes)	-0.16	0.68	-0.24	0.58
Interaction between palatability treatment and prior DB exposure (DB*yes)	0.30	0.96	0.31	0.75

Significant *P*-values (when present) are shown in bold.

In examining if prior exposure to DB made spiders more wary (i.e., slower) to attack DB termites, we found that spiders exposed to 1% DB tended to be slower to attack the DB-treated termites (interaction between palatability treatment and prior DB exposure (with naive spiders attacking control termites as reference): -658.17 ± 386.92 seconds, *t* = -1.70, *P* = 0.09). However, this non-significant trend did not replicate in the experiment with a higher concentration of DB (3%) where a stronger effect would have been expected (interaction between palatability treatment and prior DB exposure: 110.37 ± 135.49 seconds, *t* = 0.82, *P* = 0.42). Since this trend did not replicate, we do not discuss it further. Regardless of prior DB exposure, there were no differences in the time it took spiders to attack control vs. DB termites (experiments with both naïve and experienced spiders: 1% DB: 188.5 ± 131.9 seconds, Control: 490.3 ± 143.0 seconds, *t* = -1.55, *P* = 0.12; 3% DB: 92.46 ± 42.42 seconds, Control: 101.77 ± 52.79 seconds, *t* = -0.14, *P* = 0.89). Collectively, this suggests that, as with naive spiders, experienced spiders were not avoiding any odors associated with the DB treatment, even after being given the opportunity to learn to associate them with unpalatability.

Overall, in these experiments with half of the spiders exposed to DB prior to testing, all spiders attacked a prey during their first trial and therefore none (0% of the 179 spiders) were retested (6 spiders were replaced due to meeting exclusion criteria defined *a priori*).

#### 1.4 Is DB effective at making termites unpalatable to naïve spiders?

**Fig 6:**
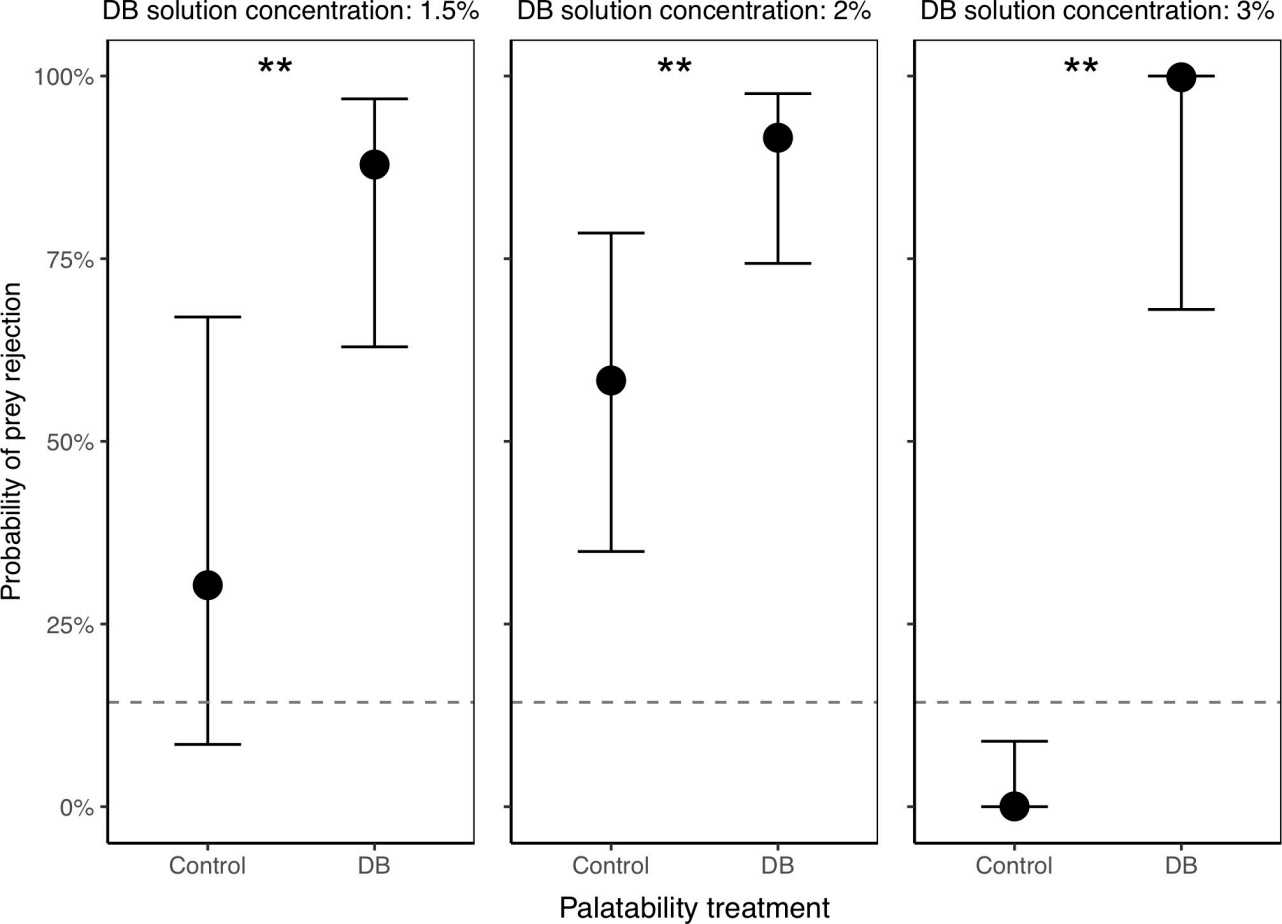
Probability of control and DB termites being rejected by spiders, in Experiment A with naïve spiders only. Across all DB concentrations tested, DB termites were significantly more likely to be rejected compared to controls, suggesting that our DB manipulation is indeed effective at making termites unpalatable. Plotted are the back-transformed model estimates with their 95% confidence intervals. The dotted line represents the baseline prey rejection rate of unmanipulated termites (estimated from a separate experiment with no DB present). A total of n = 90 spiders were tested (n = 30 at each of the three DB concentrations). Asterisks indicate significant differences between control and DB termites (*P<0.05, **P<0.01, ***P<0.001).

In experiments with naïve spiders only (Experiment A), spiders were significantly more likely to reject DB termites than control termites across the DB concentration gradient (Fig 6 and Table 3), suggesting that DB is effective at making termites unpalatable. As expected, the color of the termites (green vs. brown) had no significant effect on the likelihood of them being rejected by the spiders (Table 3).

**Table 3:** Model estimates (±SE) of the probability of termites being rejected after attack, in Experiment A with naive spiders only. Values are relative to the intercept which represents brown control termites. *Z* and *P* values are indicated for each fixed effect. The significant effect of palatability treatment at all three DB concentrations indicates that the DB treatment was effective at making termites unpalatable to spiders.

Factor	Estimate	±SE	z	*P*
Experiment with 1.5% DB (n = 30)				
Intercept	-0.83	0.79	-	-
color (green)	-1.39	0.76	-1.83	0.07
palatability treatment (DB)	2.81	0.89	3.18	**0.001**†
Experiment with 2% DB (n = 30)				
Intercept	0.34	0.49	-	-
color (green)	-1.05	0.62	-1.69	0.09
palatability treatment (DB)	2.05	0.68	3.01	**0.0015**†
Experiment with 3% DB (n = 30)				
Intercept	-7.74	2.77	-	**-**
color (green)	0.11	2.25	0.05	0.96
palatability treatment (DB)	14.13	4.53	3.12	**0.001**†

Significant *P*-values are shown in bold.

Daggers (†) indicate *P*-values that were divided by 2 to comply with one-tailed hypothesis testing as planned *a priori* (see Statistical Analysis section and preregistration document [46]).

Although DB termites were significantly more likely to be rejected, spiders did occasionally consume them, suggesting that individual variation in response to distastefulness (or individual differences in metabolic rate or hunger level) may play a role in rejection probability. For the experiment using 1.5% DB, out of 36 attacks on DB termites, 29 attacks led to rejection (80.6%), and 7 to consumption (19.4%). For the 2% DB experiment, out of 34 attacks on DB termites, 29 led to rejection (85.3%), and 5 to consumption (14.7%). For the 3% DB experiment, out of 26 attacks on DB termites, 21 led to rejection (80.8%), and 5 to consumption (19.2%). Note that the descriptive data here include repeated attacks by the same female (which was accounted for by a random effect in our model above).

Our data is consistent with a wide range of studies reporting that predators will repeatedly re-sample prey even after encountering those that are distasteful [83, 84]. When considering using DB in experiments, it is important to confirm that DB is not so aversive that predators exposed to it once will stop attacking prey altogether and be unmotivated to feed, but instead, will continue to participate in experiments. This is particularly important for experiments where predators are being trained over long periods and therefore need to repeatedly interact with distasteful prey.

#### 1.5 Do spiders habituate to the taste of DB?

**Fig 7:**
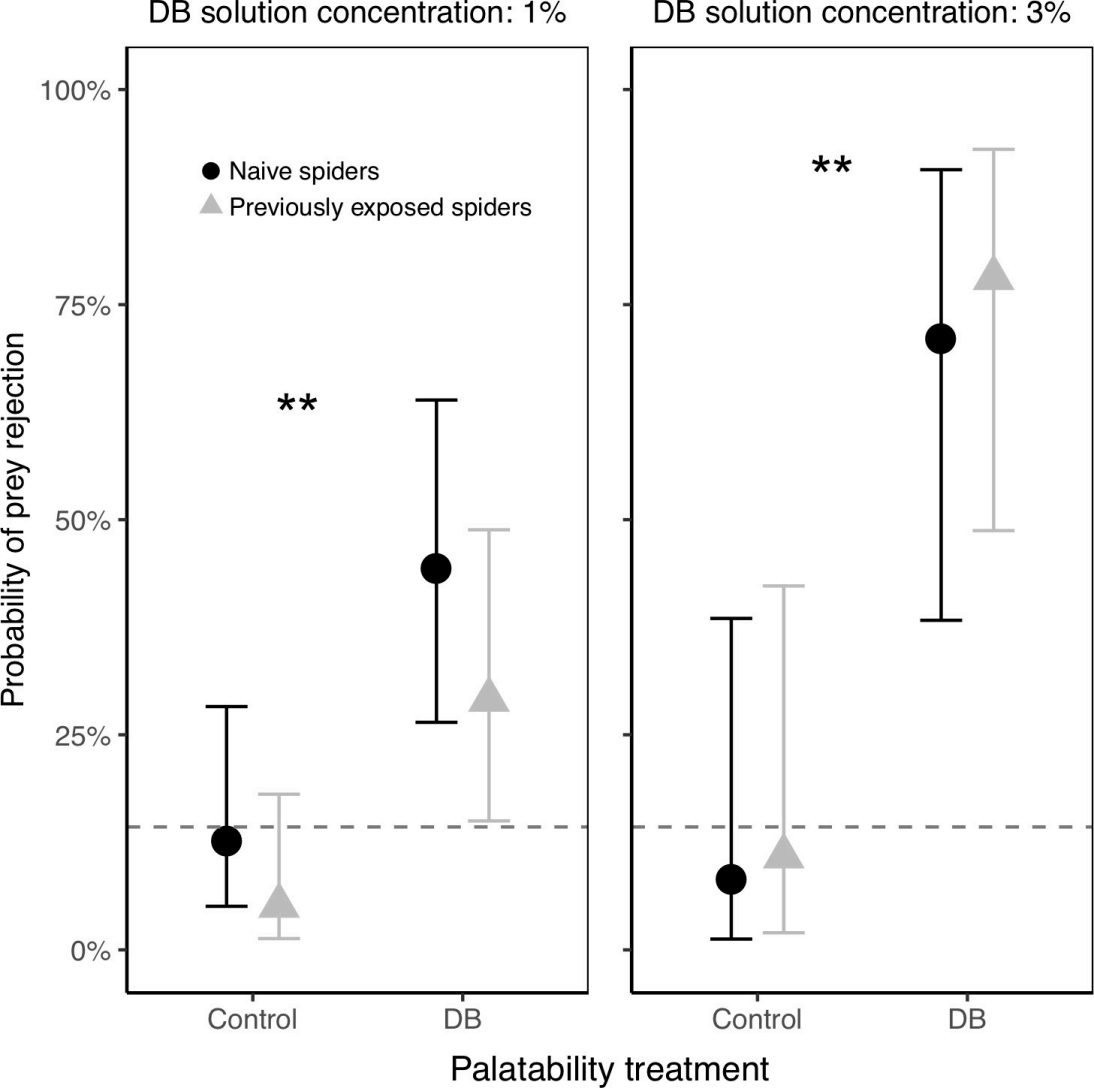
Probability of control and DB termites being rejected by spiders, in Experiment B with both naïve spiders and those that had been previously exposed to DB. Naïve and exposed spiders responded similarly to the DB treatment; regardless of prior exposure, spiders were more likely to reject DB termites compared to controls, indicating that our DB treatment was effective at making termites unpalatable. Plotted are the back-transformed model estimates with their 95% confidence intervals. The dotted line represents the baseline prey rejection rate of unmanipulated termites (estimated from a separate experiment with no DB present). A total of n = 100 spiders were used for the experiment using DB with a 1% concentration, and n = 79 spiders were used for the experiment with 3% DB. Asterisks indicate significant differences between control and DB termites (*P<0.05, **P<0.01, ***P<0.001).

In the experiments that involved both naïve spiders and those previously exposed to DB (Experiment B), spiders previously exposed to DB were not less likely than naive spiders to reject DB termites (i.e., there was no interaction between prior DB exposure and palatability treatment), suggesting that the spiders were not increasing their acceptance of DB termites with experience (Fig 7 and Table 4). Regardless of the spiders’ prior exposure, they were significantly more likely to reject DB termites compared to control termites (Fig 7 and Table 4). For the experiment at 1% DB, out of 84 attacks on DB termites, 45 attacks led to rejection (53.6%), and 39 to consumption (46.4%). For the experiment at 3% DB, out of 83 attacks on DB termites, 54 led to rejection (65.1%), and 29 to consumption (34.9%). Note, as above, that the descriptive data here include repeated attacks by the same female (which was accounted for by a random effect in our model). Unexpectedly, in one experiment (1% DB concentration), green termites were significantly more likely to be rejected than brown termites (Table 4), but this effect did not replicate in any of the other experiments where non-significant effects of color varied in direction.

**Table 4:** Model estimates (±SE) of the probability of a particular termite to be rejected after attack, in Experiment B with both naïve spiders and those that were previously exposed to DB. Values are relative to the intercept which represents naïve spiders offered brown control termites. *Z* and *P* values are indicated for each fixed effect. The lack of any significant interaction between palatability treatment and prior DB exposure suggest that both naïve and exposed spiders responded similarly to the DB treatment. The significance of the main effect of palatability treatment at both DB concentrations indicates that the DB treatment was effective at making termites unpalatable to spiders.

Factor	Estimate	±SE	z	*P*
Experiment with 1% DB (n = 100)				
intercept	-2.92	0.72	-	-
color (green)	1.27	0.40	3.17	**0.002**
palatability treatment (DB)	2.02	0.70	2.88	**0.002**†
prior DB exposure (yes)	0.98	0.73	1.35	0.18
Interaction between palatability treatment and prior DB exposure (DB*yes)	-0.32	0.86	-0.37	0.71
Experiment with 3% DB (n = 79)				
intercept	-2.42	0.99	-	-
color (green)	-1.23	0.67	-1.83	0.07
palatability treatment (DB)	3.31	1.10	3.01	**0.0015**†
prior DB exposure (yes)	0.31	1.00	0.31	0.75
Interaction between palatability treatment and prior DB exposure (DB*yes)	0.06	1.17	0.05	0.96

Significant *P*-values are shown in bold.

Daggers (†) indicate *P*-values that were divided by 2 to comply with one-tailed hypothesis testing as planned *a priori* (see Statistical Analysis section and preregistration document [46]).

In birds, it has been shown that the bitterness of DB is not a reliable indicator of toxicity due to the lack of undesirable post-ingestive effects [54]; given this, it is possible that they could dissociate the bitter taste with genuine toxicity, leading them to alter their rejection behaviors (i.e., habituate to the bitter taste) over time [14]. While we found no evidence that our spiders habituated to the taste of DB with experience, we can’t rule out the possibility that habituation would occur over a longer timescale. This is something that should be examined in future work, particularly if designing experiments that require predators to continually interact with DB-treated prey.

**1.6 Does increasing DB concentration increase its effectiveness at making termites unpalatable to spiders?** The rejection rate of unpalatable termites across all experiments showed a significant increase with DB concentration, followed by a decline (or at least plateau) at high concentrations (quadratic term: *z* = -3.21, *P* = 0.001, S3 Fig).

These data allow us to speculate that a subtle gradient of concentrations could be used to vary the level of unpalatability (perhaps to scale with a gradient of color for studies of aposematism) but more work needs to be done to validate this in more detail.

**1.7 Is there a risk of contamination between DB-treated and control termites when used simultaneously in an experiment?** The baseline rejection rate of unmanipulated termites (i.e., the rate at which spiders will drop or mishandle unmanipulated termites after they attack them) was 14.3% when no source of DB contamination was possible (indicated by dotted lines in Figs 6 and 7). Across all of our DB-manipulation experiments, we found variable rejection rates for control termites that were sometimes unexpectedly higher than the baseline rejection rate (see Figs 6 and 7). This suggests the possibility that control termites (supposedly free of DB) were sometimes unintentionally getting contaminated with low levels of DB from physical interactions with the DB-treated termites during our tests.

Any possible contamination issues did not seem to be driven by the mouthparts or legs of the spiders themselves getting contaminated with DB, as control termites which were attacked after the spider had first attacked a DB termite were not more likely to be rejected than control termites that were attacked first (experiments with naïve spiders (Experiment A) using 1.5% DB: odds ratio (odds of the control termite being rejected when attacked after a DB termite as opposed to when attacked first) = 1.93, 95% confidence interval (CI) = 0.12–24.70, *P* = 0.60; 2% DB: odds ratio = 0.33, CI = 0.01–4.25, *P* = 0.61; 3% DB: odds ratio = 1.19, CI = 0.02–28.18, *P =* 1.00; experiments with naïve and DB-exposed spiders (Experiment B) using 1% DB: odds ratio = 0.35, CI = 0.01–3.00, *P* = 0.43; 3% DB: odds ratio = 1.99, CI = 0.25–14.21, *P* = 0.40).

These data indicate that contamination is possible in experiments, and in some cases may be unavoidable. Even low levels of contamination are important to consider as it could influence the outcome and interpretation of an experiment. To mitigate possible contamination issues in future studies (if needed), treated and control prey could be offered sequentially, or in separate containers [85] where they can’t make physical contact with one another during experiments. Additionally, we strongly advise handling DB and the laboratory instruments that come into direct contact with it cautiously, including spraying prey outdoors or under a fume hood. It is important to note that regardless of the contamination in our study, our data suggests that this method still works well enough to create categories of prey that differ in palatability.

### Experimental set 2: Milkweed-induced unpalatability in bugs

#### 2.1 Do our color and palatability manipulations unintentionally influence bug movement rate?

Milkweed-fed bugs that were painted and sunflower-fed bugs that were unpainted had similar movements rates to milkweed-fed bugs that were left unpainted (z = 1.13, *P* = 0.26 and z = -1.62, p = 0.12, respectively); this suggests that neither our color manipulation methods (including our method of anesthesia) nor diet manipulation unintentionally altered this aspect of prey behavior (S4 Fig). As described previously, prey movement often elicits attacks from jumping spiders [79, 80], and therefore it was important to rule out such differences in behavior.

Anecdotally, we noticed increased grooming behavior for the first few minutes after painting occurred (which stopped before our testing began), so we would recommend that researchers using this method wait at least 10 minutes before using painted bugs in experiments. Furthermore, we would recommend using painted bugs within 24 hours after being painted as these bugs die after 2 or 3 days, possibly because the paint is obstructing their spiracles.

#### 2.2 Are there aversive odors associated with unpalatable milkweed-fed bugs (compared to sunflower-fed bugs) that spiders can detect prior to attack?

**Fig 8:**
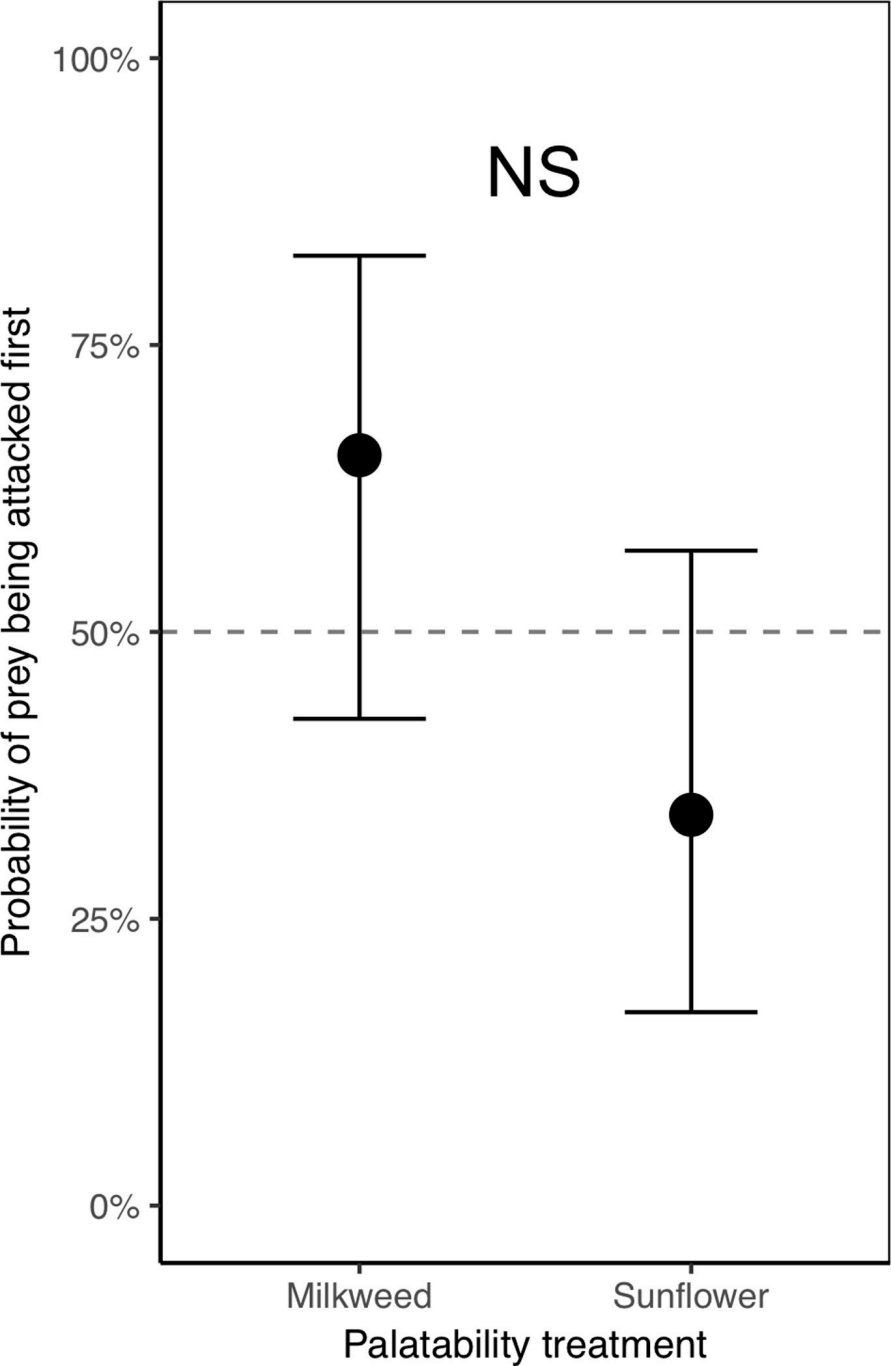
Probability of milkweed and sunflower bugs being attacked first in a trial, in Experiment C where all spiders had prior exposure to milkweed bugs. The lack of a significant difference in the probability of attack on milkweed vs. sunflower bugs suggests that there are no aversive odors produced by the milkweed bugs that deter spider attack. The dotted line at 50% is shown for reference as the expected probability of attack for each group if spiders were not differentiating between milkweed and sunflower bugs. Plotted are the back-transformed model estimates with their 95% confidence intervals. A total of n = 40 spiders were tested. NS indicates no significant differences between groups.

There was no significant difference in the probability of attack directed towards MW vs. SF bugs (trend opposite expectation with slightly more attacks on MW bugs, Fig 8 and Table 5), suggesting that there are no aversive odors associated with MW bugs that deter attack (Experiment C). As expected, color (green vs. brown painted) had no effect on attack likelihood (see Table 5).

**Table 5:** Model estimates (±SE) of the probability of a particular bug being attacked first, in Experiment C where all spiders had prior exposure to milkweed bugs. Values are relative to the intercept which represents brown milkweed bugs. *Z* and *P* values are indicated for each fixed effect. The lack of a significant effect of palatability treatment suggests that spiders were not biasing their attacks away from milkweed bugs, as would be expected if the presence of unpalatable milkweed toxins had an aversive odor that deterred spider attack. Note that there is a non-significant trend in the opposite direction, where spiders are more, rather than less, likely to attack milkweed-fed bugs.

Factor	Estimate	±SE	z	*P*
Experiment with bugs (n = 40)				
intercept	1.14	0.64	-	-
color (green)	-0.93	0.71	-1.31	0.20
palatability treatment (SF)	-1.34	0.71	-1.88	0.06

Significant *P*-values (when present) are shown in bold.

Spiders took the same amount of time prior to attacking MW and SF bugs (MW: 1171.1 ± 273.3 seconds, SF: 523.4 ± 372.4 seconds, t = -1.4, p = 0.17), further suggesting that the MW bugs have no aversive odor.

These results are interesting because milkweed-fed bugs have a distinctive odor (that we could smell) that is different than the smell of SF bugs, and previous work has shown that jumping spiders can learn to avoid the aversive odor associated with a closely related prey species [22]. As with the results of the DB-treated termite tests, the lack of attention to any odors associated with the bugs in our experiment could be explained in a few ways: it could be that the spiders could not detect any odors, or that they could detect them but did not respond to them (perhaps because they were focused more on visual cues), or that the enamel paint from our color manipulation masked odor-releasing glands (a technique that has been successfully implemented with other insects, see [86]). Regardless of the reason, the fact that spiders do not differentiate between the two groups of bugs makes this a useful method for manipulating palatability.

Overall, attack rates from these spiders (which were all trained on unpainted MW bugs) were lower than for any of the termite tests: only 52.5% of the spiders attacked a prey during their first test while 47.5% (vs. 2% of the 299 spiders tested with termites) of the spiders needed to be retested (spiders experienced between 1 and 5 tests, median = 1) as they had not made a single attack within the specified 2 hour period (all spiders participated in the experiment by the 4th retest, so none were discarded). We suspect that visual cues other than color (e.g., prey morphology) were used for prey recognition [87] and that previous negative experiences with these highly unpalatable bugs might have made them reluctant to attack any type of bugs (which besides their differences in palatability, are visually identical). However, despite lower attack rates on the bugs, they do attack and re-attack them enough (particularly when hungry) that they have proven to be useful experimental tools [20, 67, 70].

**Fig 9:**
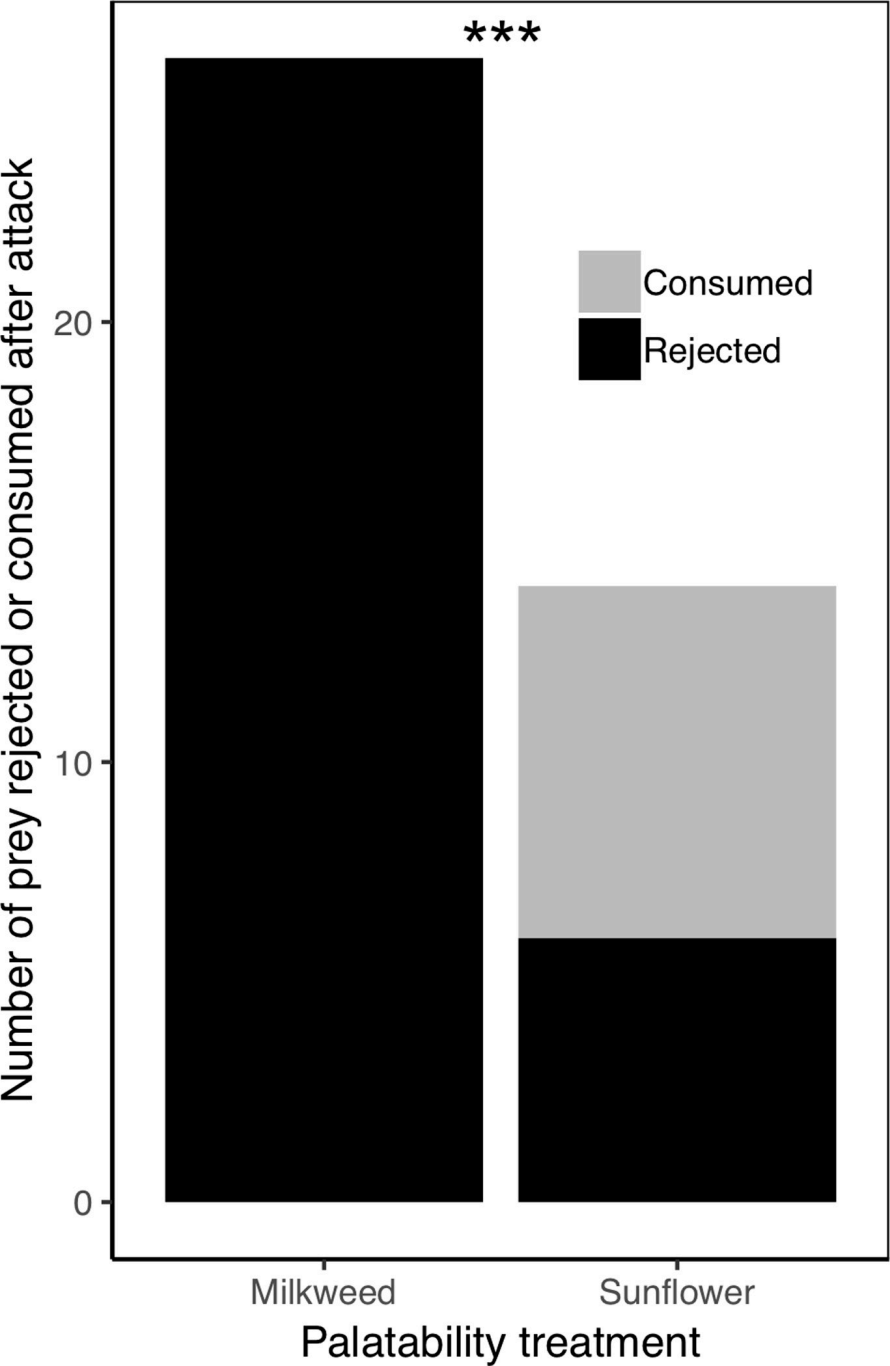
Number of milkweed and sunflower bugs rejected or consumed after attack, in Experiment C where all spiders had prior exposure to milkweed bugs. The higher rejection rate of milkweed bugs compared to sunflower bugs indicates that the milkweed diet is effective at reducing palatability of these bugs to spiders. Note that plotted data include only the first attack by each spider (as these were the data used in our statistical test to avoid pseudoreplication). Asterisks indicate significant differences between treatment groups (*P<0.05, **P<0.01, ***P<0.001).

**2.3 Is a milkweed diet effective at making bugs unpalatable to experienced spiders?** As expected, MW bugs were significantly more likely to be rejected than SF bugs (*P*<0.001, MW bugs were always rejected, so odds ratio and CI could not be calculated, Fig 9); this result confirms that the MW bugs were indeed less palatable than the SF bugs and is consistent with previous work [20, 67, 68, 70].

MW bugs were never consumed (out of 41 total attacks), while SF bugs were consumed at a rate of 60% (25 total attacks with 15 consumptions); note that this summary data includes all bug attacks including multiple attacks from the same spider within a trial, while our statistical analyses for this question only include the first attack from each spider to avoid pseudoreplication (see Statistical Analysis section). Color had no effect on prey rejection (odds of rejecting a brown bug as opposed to a green one was 1.64 (CI = 0.26–10.67, *P* = 0.69)).

Given that many animals experience detrimental post-ingestive effects after consuming insects sequestering milkweed cardenolides [88, 68] (unlike with DB), distastefulness in this case serves as an honest signal for toxicity. Our results here show a relatively high rejection rate of palatable SF bugs (in comparison to palatable termite rejection rates, see above), which was surprising considering SF bugs do not sequester any toxins [89]. We suspect generalized aversions to bugs (described previously) and mishandling (due to more cumbersome prey) contributed to this finding. Many other hemipterans also sequester toxins [90], have similar morphology as nymphs, and can be painted in a similar fashion, and therefore it is likely that similar methods of diet and color manipulation can be used effectively with a variety of hemipteran species.

### Experimental set 3: Application of DB treatment to other prey types

#### 3.1 Is DB effective when applied to other types of color-manipulated prey?

**Fig 10:**
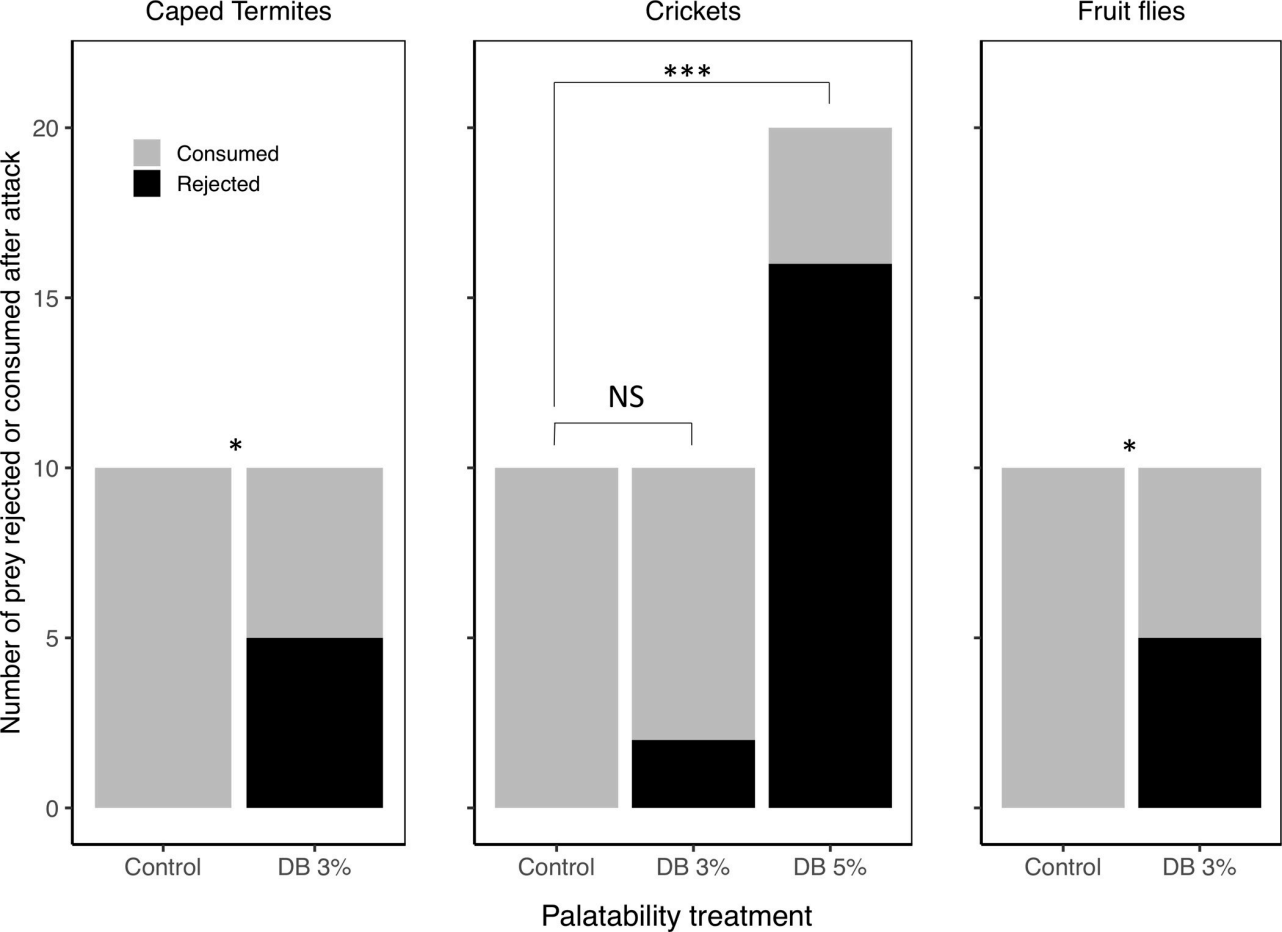
Number of DB or control prey rejected or consumed after attack, in Experiment D where spiders were presented with a single prey item at a time (either control or DB). For both caped termites and fruit flies, the higher rejection rate of DB-treated prey indicates that the treatment effectively reduces the palatability of these prey to spiders. For crickets, our 3% DB solution was not effective at reducing palatability, but an increase to 5% DB was effective. Asterisks indicate significant differences between treatment groups (*P<0.05, **P<0.01, ***P<0.001).

Generalist predators routinely encounter diverse prey types, and thus we aimed to develop a method that was versatile enough to be used with a variety of small prey species. When a 3% DB solution was applied to paper-caped termites, spiders attacked and rejected DB caped termites at a higher rate than sham-treated controls (odds ratio could not be calculated because control caped termites were never rejected; *P* = 0.02, n = 20 spiders, Fig 10). Considering all attacks made overall (even repeated attacks by the same spiders), out of 26 recorded attacks on DB caped termites, 19 (73.08%) led to rejection of the prey.

When a 3% DB solution was applied to red-dyed crickets, spiders did not reject these DB crickets at a higher rate than sham-treated control crickets (odds ratio could not be calculated because control crickets were never rejected, *P* = 0.24, n = 20 spiders, Fig 10). Overall, out of 12 attacks, 4 (33.33%) let to rejection. We suspected that the ineffectiveness of the DB treatment in this case was because the crickets actively groomed off the DB, which commonly occurs in response to noxious stimuli [91, 92]). Alternatively, differences in surface area to body mass ratio might have rendered DB less effective with crickets. When we increased the DB concentration to 5%, the treatment was more effective; spiders were more likely to reject the DB treated crickets compared with the same sham-treated controls (*P*<0.001, Fig 10). The overall prey rejection rate of the 20 spiders newly tested was 69.44% (out of 36 attacks, 25 were rejected). This suggests that specific concentrations of DB might have to be worked out for individual prey species, particularly those that are prone to grooming.

When a 3% DB solution was applied to red-eyed *Drosophila*, spiders attacked and rejected DB-treated flies at a higher rate than sham-treated controls (odds ratio could not be calculated because control flies were never rejected, *P* = 0.02, n = 20 spiders, Fig 10). Overall, out of 13 attacks on DB-treated flies, 8 (61.5%) led to rejection.

Overall, all spiders attacked the single prey presented on their first trial (sometimes even multiple times) and therefore no spider was retested or excluded. Collectively, our results suggest that DB can be applied to a variety of different live prey types. Considering DB is effective when applied externally, it would also likely be an effective tool if applied to lures or artificial prey models, which have been previously used with jumping spiders [93, 87], further expanding the potential applications of this method.

## Summary and conclusions

In this study, we show that denatonium benzoate (DB) solution, as well as sequestered milkweed toxins that can be manipulated in the diet, are effective tools for manipulating the palatability of small live insect prey (termites, milkweed bugs, and fruit flies) in experiments when using *H*. *pyrrithrix* jumping spiders as predators. Moreover, these palatability manipulations can be used effectively in conjunction with various color manipulations that we described. Our data show that that prey movement rate was not altered by our palatability or color manipulations, although we recommend comparing groups that differ in only one variable at a time when making comparisons in an experiment (e.g., painted sunflower vs. painted milkweed, or unpainted sunflower vs. unpainted milkweed). Across 419 predation trials that tested 229 individual spiders, we found that spiders did not discriminate between palatability-manipulated and control prey prior to attack (i.e., odors from our palatability manipulations were either not conspicuous or ignored) even after the spiders were repeatedly exposed to them. Moreover, the palatability manipulations were successful; across multiple experiments, treated prey were consistently more likely to be rejected after capture than control prey. While we found evidence of low-level contamination in one of our DB experiments, these manipulations were still effective, and we discussed ways to mitigate such contamination.

While previous learning studies have used DB-treated prey [42, 54, 61] and diet-manipulated milkweed bugs [25, 69] as aversive stimuli, here we scaled down these methods for use with smaller, understudied arthropod predators and thoroughly investigated the merits (and potential drawbacks) for each method. Our data therefore provide practical guidance for using these techniques effectively in future manipulative experiments that examine learning, foraging, and aposematism. A particularly exciting area of research that has received much recent attention is that of multimodal warning displays [94, 95]. To study multimodal warning displays, we need more experiments that independently manipulate color and palatability, alongside other display components (odors, sounds, etc.); we hope that our study will encourage more research in this area, particularly with small invertebrate predators and prey. Due to the striking diversity of sensory capabilities in many predators, the recent emphasis surrounding complex signals is not surprising, and we argue that as behavioral ecologists, we should continue to broaden our toolkit in ways that allow us to study multiple predator and prey taxa.

## Supporting information

S1 Fig Spectral properties of the green and brown paint used to artificially manipulate prey color (both termites and bugs) in prey choice tests. Spectral curves represent mean values for 10 measurements of each color.
(TIFF)

S2 Fig Movement rates of control and DB-treated termites (estimated by the number of gridlines crossed in the test arena) during a two-minute period. The lack of a significant difference between control and DB termites (treated with 3% DB concentration, the highest concentration used in our termite experiments) suggests that our DB manipulations had a negligible effect on termite movement rate. Plotted are the back-transformed model estimates with their 95% confidence intervals. A sample size of n = 120 termites was used, with an equal number of control and DB termites painted brown or green (including all possible combinations of color and treatment). NS denotes no significant difference between control and DB termites.
(TIFF)

S3 Fig Probability for a DB termite to be rejected as a function of the concentration of DB used, across all of our termite experiments. The likelihood of a spider rejecting a DB termite initially increases with increasing DB concentration and then decreases/plateaus. Plotted are the back-transformed model estimates with their 95% confidence intervals, using all attacks in all termite experiments. Sample sizes of n = 100, n = 30, n = 30, and n = 109 spiders were used for concentrations 1%, 1.5%, 2%, and 3%, respectively.
(TIFF)

S4 Fig Movement rates of painted milkweed bugs, unpainted milkweed bugs, and unpainted sunflower bugs (estimated by the number of gridlines crossed in a test arena) during a two-minute period. The lack of significant differences between painted and unpainted milkweed bugs suggests that our color manipulation did not unintentionally alter bug movement rate. The lack of significant differences between unpainted milkweed bugs and unpainted sunflower bugs suggests that the diet manipulation does not alter prey movement rate. Squares are the back-transformed model estimates with their 95% confidence intervals. Sample sizes of n = 20 were used for each group. NS indicates no significant differences between groups.
(TIFF)
