# Supplementary figures and images for: Methods for independently manipulating palatability and color in small insect prey
Source: PLoS One. 2020 Apr 7;15(4):e0231205. doi: 10.1371/journal.pone.0231205 (PMC7138310; doi:10.1371/journal.pone.0231205)

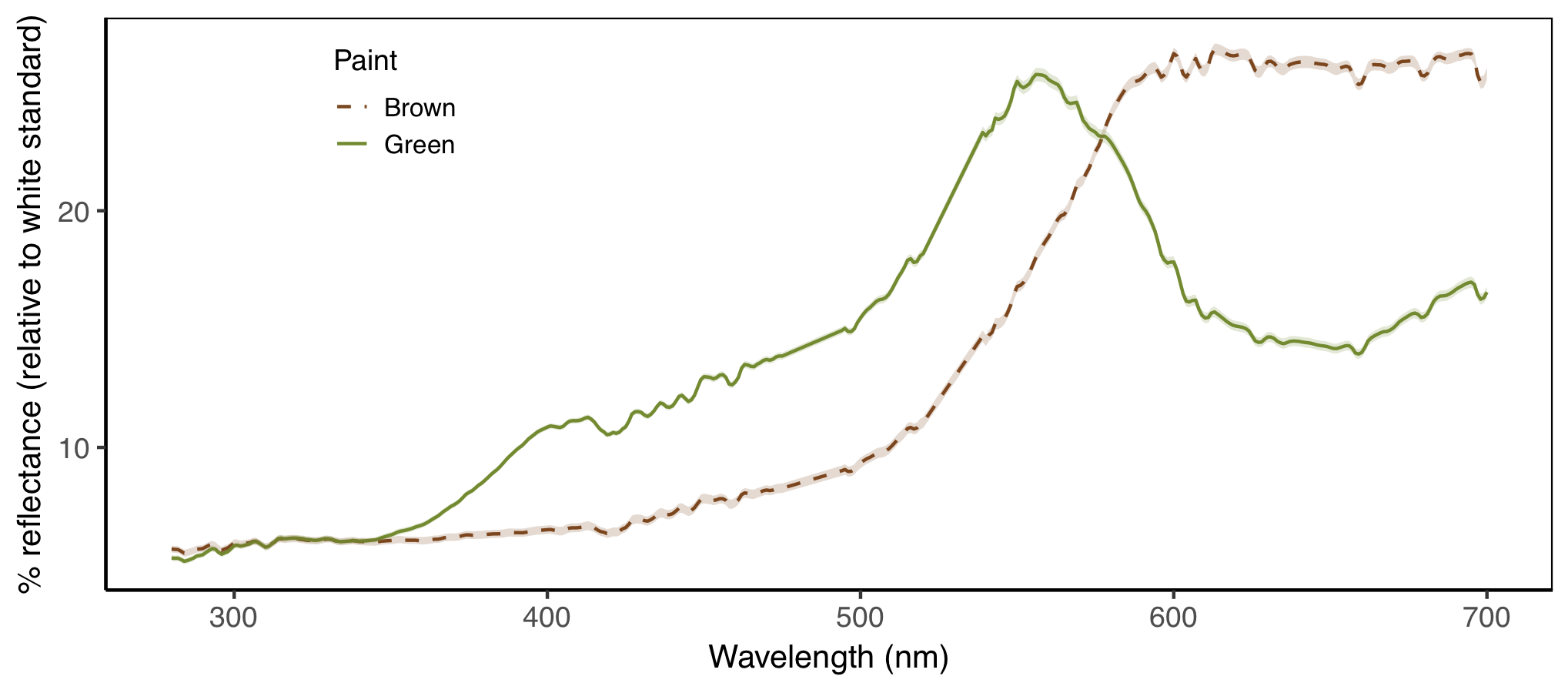

Supplement: S1 Fig — Spectral curves represent mean values for 10 measurements of each color. (TIFF) [file pone.0231205.s001.tiff]

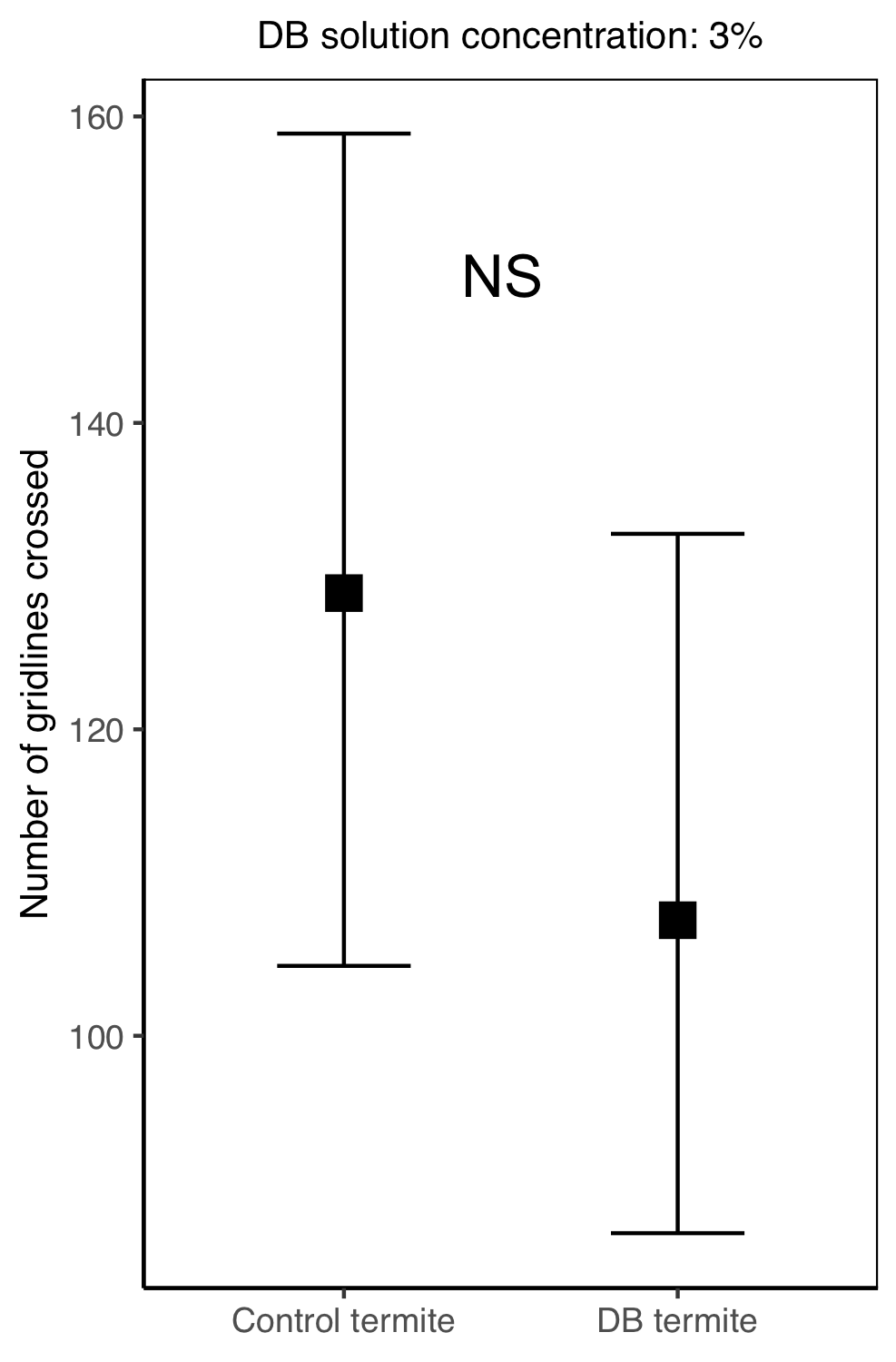

Supplement: S2 Fig — The lack of a significant difference between control and DB termites (treated with 3% DB concentration, the highest concentration used in our termite experiments) suggests that our DB manipulations had a negligible effect on termite movement rate. Plotted are the back-transformed model estimates with their 95% confidence intervals. A sample size of n = 120 termites was used, with an equal number of control and DB termites painted brown or green (including all possible combinations of color and treatment). NS denotes no significant difference between control and DB termites. (TIFF) [file pone.0231205.s002.tiff]

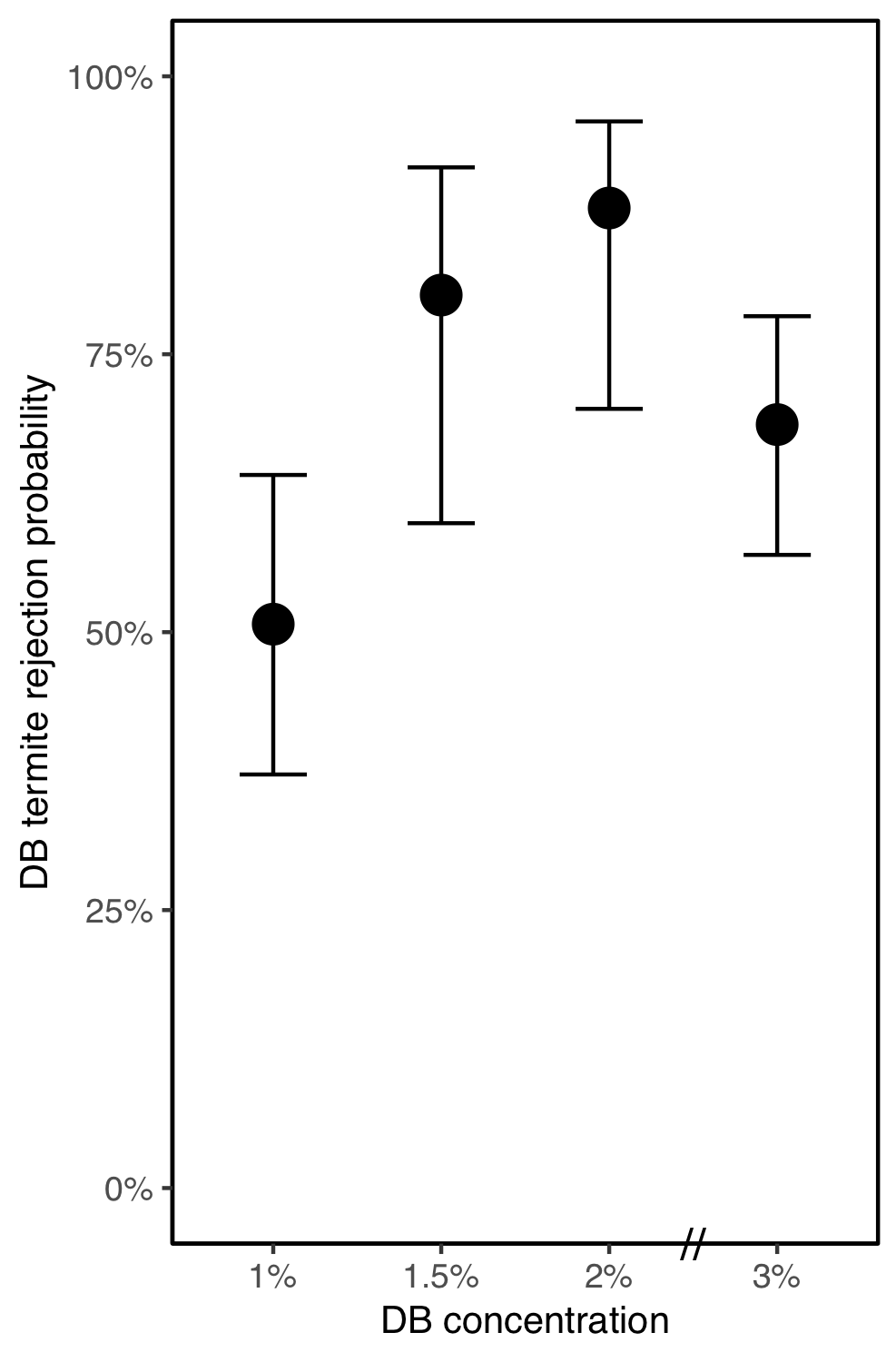

Supplement: S3 Fig — The likelihood of a spider rejecting a DB termite initially increases with increasing DB concentration and then decreases/plateaus. Plotted are the back-transformed model estimates with their 95% confidence intervals, using all attacks in all termite experiments. Sample sizes of n = 100, n = 30, n = 30, and n = 109 spiders were used for concentrations 1%, 1.5%, 2%, and 3%, respectively. (TIFF) [file pone.0231205.s003.tiff]

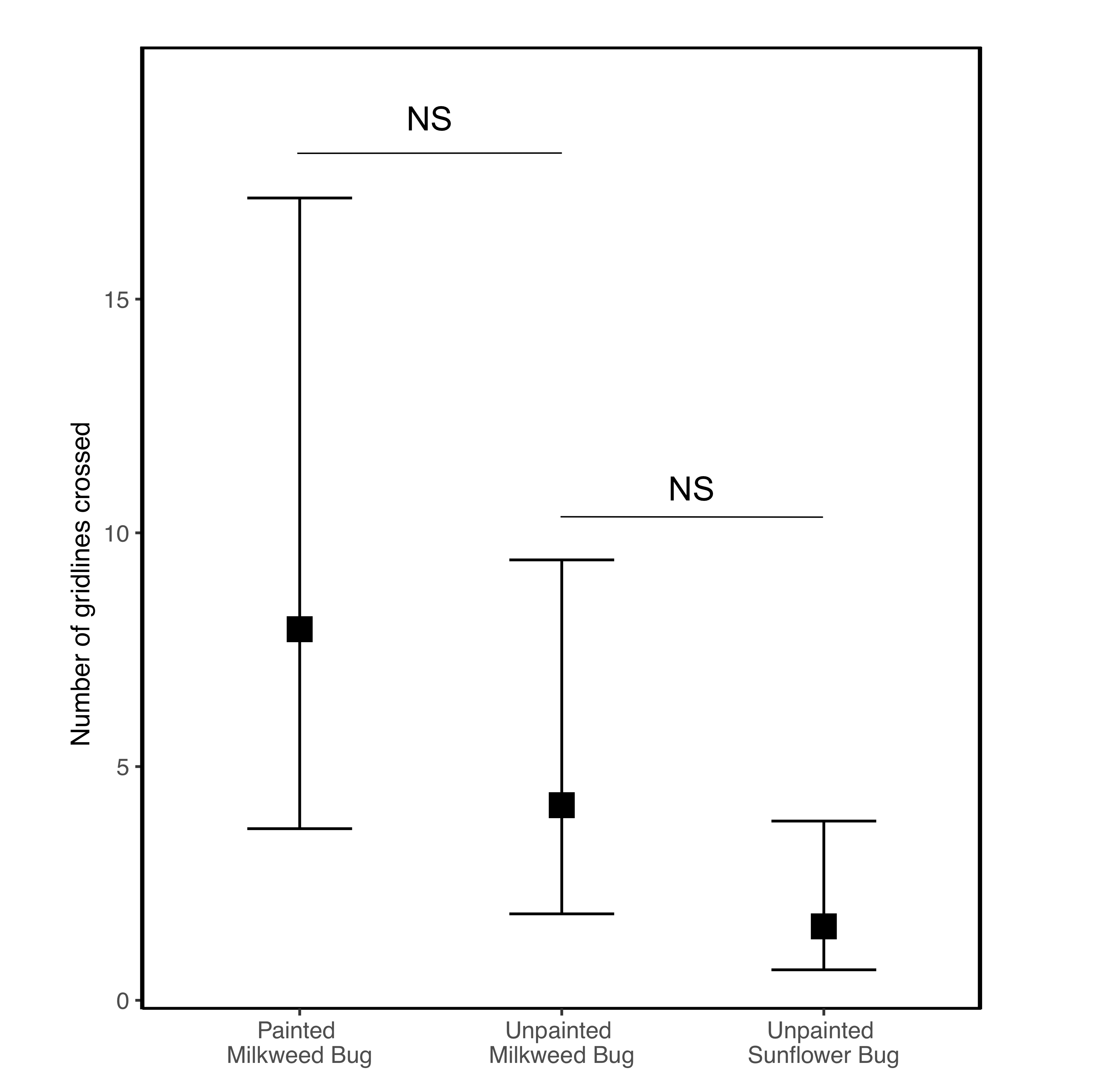

Supplement: S4 Fig — The lack of significant differences between painted and unpainted milkweed bugs suggests that our color manipulation did not unintentionally alter bug movement rate. The lack of significant differences between unpainted milkweed bugs and unpainted sunflower bugs suggests that the diet manipulation does not alter prey movement rate. Squares are the back-transformed model estimates with their 95% confidence intervals. Sample sizes of n = 20 were used for each group. NS indicates no significant differences between groups. (TIFF) [file pone.0231205.s004.tiff]
